# Interplay of dFOXO and Two ETS-Family Transcription Factors Determines Lifespan in *Drosophila melanogaster*

**DOI:** 10.1371/journal.pgen.1004619

**Published:** 2014-09-18

**Authors:** Nazif Alic, Maria E. Giannakou, Irene Papatheodorou, Matthew P. Hoddinott, T. Daniel Andrews, Ekin Bolukbasi, Linda Partridge

**Affiliations:** 1 Institute of Healthy Ageing, and GEE, University College London, London, United Kingdom; 2 EMBL - European Bioinformatics Institute, Wellcome Trust Genome Campus, Hinxton, Cambridge, United Kingdom; 3 Max Planck Institute for Biology of Ageing, Cologne, Germany; Stanford University Medical Center, United States of America

## Abstract

Forkhead box O (FoxO) transcription factors (TFs) are key drivers of complex transcriptional programmes that determine animal lifespan. FoxOs regulate a number of other TFs, but how these TFs in turn might mediate the anti-ageing programmes orchestrated by FoxOs *in vivo* is unclear. Here, we identify an E-twenty six (ETS)-family transcriptional repressor, *Anterior open* (*Aop*), as regulated by the single *Drosophila melanogaster* FoxO (dFOXO) in the adult gut. AOP, the functional orthologue of the human Etv6/Tel protein, binds numerous genomic sites also occupied by dFOXO and counteracts the activity of an ETS activator, *Pointed* (*Pnt*), to prevent the lifespan-shortening effects of co-activation of dFOXO and PNT. This detrimental synergistic effect of dFOXO and PNT appears to stem from a mis-regulation of lipid metabolism. At the same time, AOP activity in another fly organ, the fat body, has further beneficial roles, regulating genes in common with *dfoxo*, such as the secreted, non-sensory, odorant binding protein (*Obp99b*), and robustly extending lifespan. Our study reveals a complex interplay between evolutionarily conserved ETS factors and dFOXO, the functional significance of which may extend well beyond animal lifespan.

## Author Summary

Despite the apparent complexity of ageing, animal lifespan can be extended. Activity of Forkhead Box O (FoxO) transcription factors can prolong survival of organisms ranging from the budding yeast to the fruit fly, and *FoxO* gene variants are linked to human longevity. FoxOs extend lifespan by driving complex, widespread changes in gene expression. Their primary targets include a second tier of transcriptional regulators, but it remains unclear how these secondary regulators are involved in the anti-ageing programmes orchestrated by FoxOs *in vivo*. To elucidate the role of this second tier, we identify a transcription factor called *Anterior open* (*Aop*) as directly regulated by the single *Drosophila melanogaster* FoxO protein (dFOXO) in the adult fly gut. Under certain circumstances, such as co-activation of the Pointed (PNT) transcription factor, dFOXO can be detrimental to lifespan. The role of *Aop* is to protect from this negative synergistic effect. Additionally, activation of AOP in the fly adipose tissue can robustly extend lifespan. Our study reveals a complex interplay between two evolutionarily conserved transcriptional regulators and dFOXO in lifespan. This significance of this interplay may extend to other physiological processes where these transcription factors play important roles.

## Introduction

Forkhead Box O (FoxO) transcription factors (TFs) play a key, evolutionarily conserved role in ageing. *Drosophila melanogaster* has a single FoxO orthologue (*dfoxo*) and increasing its activity in certain tissues is sufficient to extend fly lifespan [1]–[4] Furthermore, both *dfoxo* and the *Caenorhabditis elegans* othologue, *daf-16*, are strictly required for lifespan extension upon reduction in insulin/IGF-like signalling (IIS) [5], [6]. This evolutionary conservation appears to extend all the way to yeast on one side, where forkhead-like factors can extend lifespan [7], and to humans on the other, where certain variants of the *FoxO3A* locus are robustly correlated with longevity [8]–[12].

FoxOs control a plethora of traits at both organismal and cellular levels, including control of cell cycle, cell death, growth and metabolism. In all cases, FoxOs can be viewed as acting to preserve homeostasis [13]. Indeed, numerous processes are remodelled by activation of FoxOs, through regulation of a large number of direct and indirect targets, all acting in concert to preserve homeostasis in old age and extend animal lifespan [14]–[19].

Several studies have examined the targets of FoxOs. A striking finding of these studies is that FoxOs control a range of other cellular regulators. These include secreted endocrine factors, components of intracellular signalling pathways and several TFs [14], [16]–[20]. Transcriptional feedback within the signalling pathway plays a role [21], but in most cases the functions of these other regulators remain unknown, both in isolated cells and, more importantly, *in vivo*.

The putative roles of TFs regulated by FoxOs are particularly intriguing. Numerous studies have shown that FoxOs interact with a number of unrelated TFs, in a number of ways, with important consequences for the output of both interacting partners [13], [22]. These TFs include Myc, p53, Smads, ß-catenin, and numerous nuclear hormone receptors [22]–[27]. Hence, there is a potential for the TFs regulated by FoxO to profoundly alter FoxO's functional output through interactions with FoxO itself. However, it remains unclear what the role of these interactions is in the whole animal, *in vivo* and, specifically, what is their role in lifespan?

In this study we set out to elucidate the role played in lifespan by a TF directly regulated by dFOXO. We identify an E-twenty six (ETS) - family transcriptional repressor, *Anterior open* (*Aop*), as regulated by dFOXO in the adult *Drosophila* gut. *Aop* is the functional orthologue of the human *Etv6* gene and, in *Drosophila*, it is known to counteract the activity of an ETS activator, *Pointed* (*Pnt*). We show that *Aop* acts to prevent the detrimental effects of co-activation of dFOXO and PNT in adult *Drosophila* gut, and we present evidence that this interaction is mediated by binding to the same genomic locations as dFOXO. AOP activation on its own in the adult fat body can also robustly extend lifespan. Our study reveals a complex interplay between evolutionarily conserved ETS-family TFs and dFOXO in longevity. The significance of this interplay may extend to other physiological processes.

## Results

### dFOXO regulates distinct genes but similar functions in the adult gut and fat body

dFOXO, like its mammalian orthologues, controls gene expression in a tissue-specific manner [19], [28]–[30]. Hence, to investigate the functional interplay between dFOXO and one of its target TFs, we turned our attention to a tissue-specific, adult-inducible, lifespan-relevant system. Over-expression of *dfoxo* using the RU486-inducible, *S_1_106* Geneswitch driver [31], robustly extends lifespan [1], [4], [32]–[34]. *S_1_106* restricts *dfoxo* induction to two specific adult fly organs: the midgut and abdominal fat body (subsequently referred to as gut and fat body; **Figure S1A**) [31], the latter functionally equivalent to mammalian white adipose tissue and liver. Both have an evolutionarily conserved role in aging [35], [36], and it is currently unclear whether activation of *dfoxo* in either organ alone is sufficient to extend lifespan. For these reasons, we chose to identify the TFs regulated by dFOXO in both of these organs.

**Figure 1:**
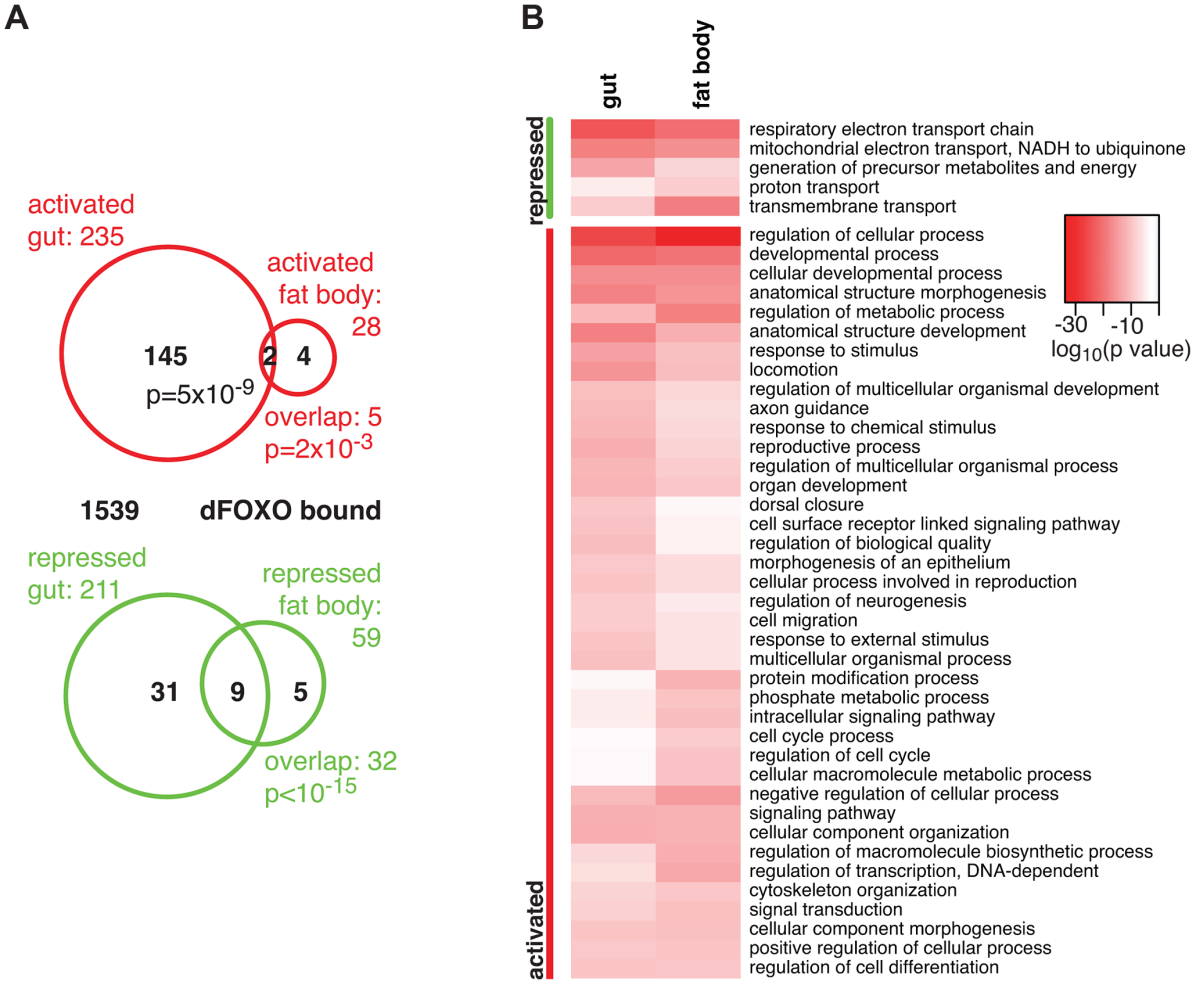
dFOXO targets in the adult gut and fat body. **A** Proportional Venn diagram showing the sets of genes that were differentially regulated by *dfoxo* induction in the gut or the fat body. The number of genes that were bound by GFP-dFOXO within each differentially expressed-gene set are given in black. p values for significant set overlaps are indicated. **B** Biological process GO categories differentially regulated (p<10^−10^) in the fat body or gut upon induction of *dfoxo* as determined by Catmap analysis. Any redundant categories (overlap by more than 75%) were removed, retaining the most specific category. The full list is given in **Dataset S1**. The intensity of red shows the log_10_-transformed p-value associated with differential regulation for each category.

We micro-dissected mid-guts or carcass-associated thoracic/abdominal fat body of *S_1_106>dfoxo* females (+/− RU486) and determined their mRNA profiles using Affymetrix gene expression arrays (ArrayExpress accession number: E-MTAB-1020). In each case, we controlled for the changes associated with induction of the driver alone (*S_1_106* +/− RU486). 447 genes were differentially expressed in the gut (p value cut-off of 0.00285 corresponding to FDR of 5%, **Figure 1A**). We detected fewer significant changes in the fat body, 87 differentially regulated genes (p value cut-off 0.0022, FDR 20%, **Figure 1A**), most-likely due to the difficulty of dissecting this loosely-associated tissue. The full list of genes regulated by dFOXO, as well as all other lists mentioned in the paper, are given in **Dataset S1**. The list included some well-known targets of dFOXO, such as *initiation factor 4E binding protein* (*4ebp*) [37], [38] and the *Drosophila insulin receptor* (*dInR*) [37], both activated in the gut, and the insulin-regulated kinase *Akt* [17], [39], induced in both the gut and fat body.

The overlap between genes regulated in the same direction in gut and fat body was significant (p = 0.002 for up-regulated, p<10^−15^ for down-regulated genes), but by no means complete (**Figure 1A**), indicating that dFOXO regulates both related and unrelated sets of genes in the two tissues. This did not appear to be caused by weaker signal obtained from the fat body, since changes unique to the fat body were also detected (e.g. *Obp99b*; see later). To further explore this, we examined the functions regulated by dFOXO in the two tissues using Catmap, an approach that can detect co-ordinated, subtle changes over many genes, rather than depending on an arbitrarily chosen, differential expression p-value cut-off [40]. Although some differences occurred, the biological process Gene Ontology (GO) categories differentially expressed in the two tissues were similar (**Figure 1B**), indicating that dFOXO regulates similar functions in the gut and in the fat body.

Notably, dFOXO strongly repressed respiratory electron transport chain components in both the gut and fat body (p = 4×10^−25^ and p = 1×10^−21^, respectively) and, in particular, components of complex I, which transfers electrons from NADH to ubiquinone (p = 2×10^−19^ and p = 2×10^−17^). Indeed, part of the effect of dFOXO on lifespan could be mediated by its repression of the components of complex I, because reducing the electron flow through this complex, by bypassing it, can extend fly lifespan [41], [42].

### 5 TFs are directly regulated by dFOXO in the adult gut

We identified a total of 16 TFs regulated by induction of *dfoxo* in either the gut or the fat body, including *p53* and the nuclear hormone receptor *HR96* (see **Dataset S1** for the full list). To further narrow down the set of interesting candidates, we isolated the TFs encoded by genes directly bound and regulated by dFOXO, thus identifying the immediate second tier of regulators. We determined the genomic regions bound by dFOXO using a GFP-dFOXO fusion protein that is functional in lifespan-extension [33]. We prepared chromatin from RU486-fed *S_1_106>GFP-dfoxo* female flies and pulled down the DNA associated with the fusion protein, and hence restricted to gut and fat body, using an anti-GFP antibody. As a control, we performed chromatin immunoprecipitation (ChIP) using the same antibody on chromatin prepared from females over-expressing *dfoxo* alone (*S_1_106>dfoxo* + RU486).

**Figure 2:**
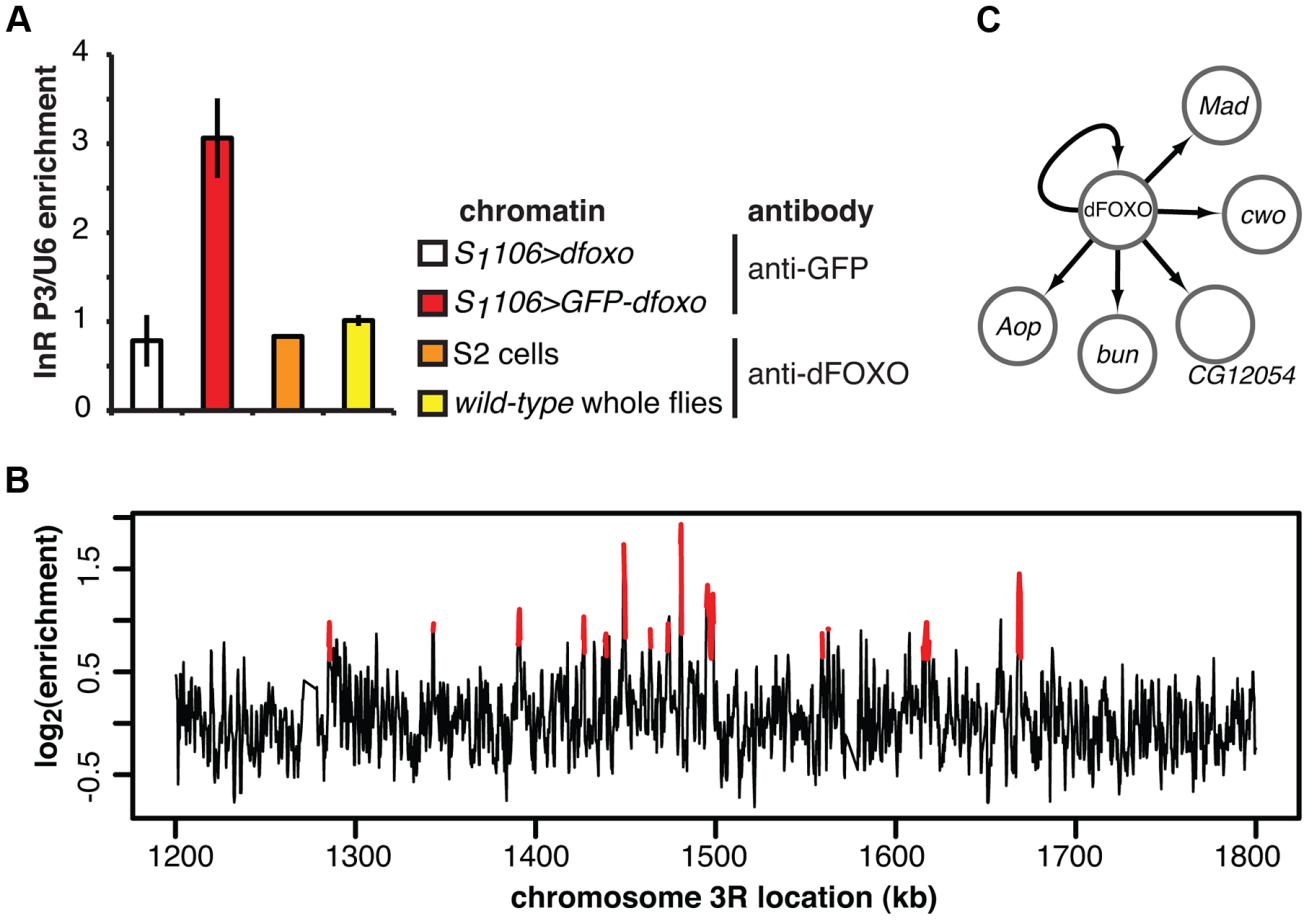
dFOXO binding sites in adult gut and fat body. **A** Enrichment of the P3 promoter sequences of the *dInR* locus in the chromatin samples prepared from RU486-fed *S_1_106>GFP-dfoxo*, RU486-fed *S_1_106>dfoxo* (mock for the anti-GFP IP), wild-type 7-day old females or 2-h serum-starved S2 cells, after IP with either anti-GFP antibody or anti-dFOXO antibody, as indicated. Enrichment is expressed relative to *U6*, as means ± SEM of three biological replicates of chromatin, except for S2 cells where three IPs were performed from the same chromatin sample. ANOVA on log-transformed data detected significant differences (p<10^−3^), and enrichment in *S_1_106>GFP-dfoxo*, after IP with anti-GFP antibody, was greater than all others (t-test, p<10^−3^). **B** ChIP-chip traces, showing the enrichment (log_2_-transformed) of the GFP-dFOXO-immunoprecipitated DNA over total chromatin, are averages of three biological repeats after subtraction of the mock and are shown over a region of chromosome 3R. Red denotes the enrichment associated with peak regions. **C** Summary of the regulatory relationships between dFOXO and the five TFs it directly induces in the adult gut. Arrows indicate transcriptional activation.

To confirm that we were detecting tissue-restricted binding, we compared the GFP-dFOXO bound sites with those that we previously identified in whole adults or S2 cells [17]. The *dInR* locus is transcribed from three promoters under tight spatio-temporal control [43]. In whole flies, dFOXO is detected as bound in the coding region of the gene and is absent from the P1 or P3 regions [17] (**Figure 2A**). The functional significance of this binding in the 3′ region in *Drosophila* is unclear but the mammalian FoxO proteins are able to act at great distances [20]. On the other hand, in serum-starved S2 cells dFOXO is bound to the P1 promoter but not the coding or P3 regions [17] (**Figure 2A**). In contrast, GFP-dFOXO expressed in the adult gut and fat body was bound to the P3 promoter of *dInR* (**Figure 2A**), revealing a different pattern of binding in these tissues.

We hybridised three biological repeats of the experimental and control ChIP samples to tiling arrays. We identified ∼1400 genomic regions bound by GFP-dFOXO in the gut and/or the fat body (ArrayExpress accession number: E-MTAB-1021; for examples of peaks identified see **Figure 2B**; for a list of all bound locations see **Dataset S1**), with highly reproducible ChIP-chip signal across the three biological replicates (**Figure S1B**). The regions occupied by dFOXO in the gut and fat body were different from those previously identified in whole flies (**Figure S1C**), further confirming that we were detecting tissue-restricted binding. They were predominantly located in the 5′ end of genes (**Figure S1D**), indicating promoter-proximal binding, in contrast to enhancer binding observed with mammalian FoxO3 [20]. The regions bound by dFOXO were enriched for forkhead-like binding motifs (**Figure S1E**), confirming conservation of *in vivo* binding-sequence preference.

Finally, we identified the genes that are likely to be directly regulated by dFOXO based on their transcriptional responsiveness to induction of *dfoxo* and proximity (<1 kb) to a GFP-dFOXO bound site (**Figure 1A**). Genes up-regulated in the gut were specifically enriched for the GFP-dFOXO bound genes (p = 5×10^−9^). This is consistent with the predominant function of dFOXO as a transcriptional activator, conserved in its worm and mammalian orthologues [16], [19], [20]. We also observed GFP-dFOXO binding in the vicinity of the genes regulated by *dfoxo* in the fat body (**Figure 1A**), but this overlap was not significant. Note that numerous GFP-dFOXO-bound sites could not be associated with specific transcriptional events. This has been observed previously for FoxO factors in flies and other organisms [16], [17], [19], [20], [28], and it is currently unclear whether this is due to technical limitations in associating expression changes to binding events, whether FoxOs detected on these sites are poised for activation under a different set of conditions, or whether some of these sites are not functional.

**Figure 3:**
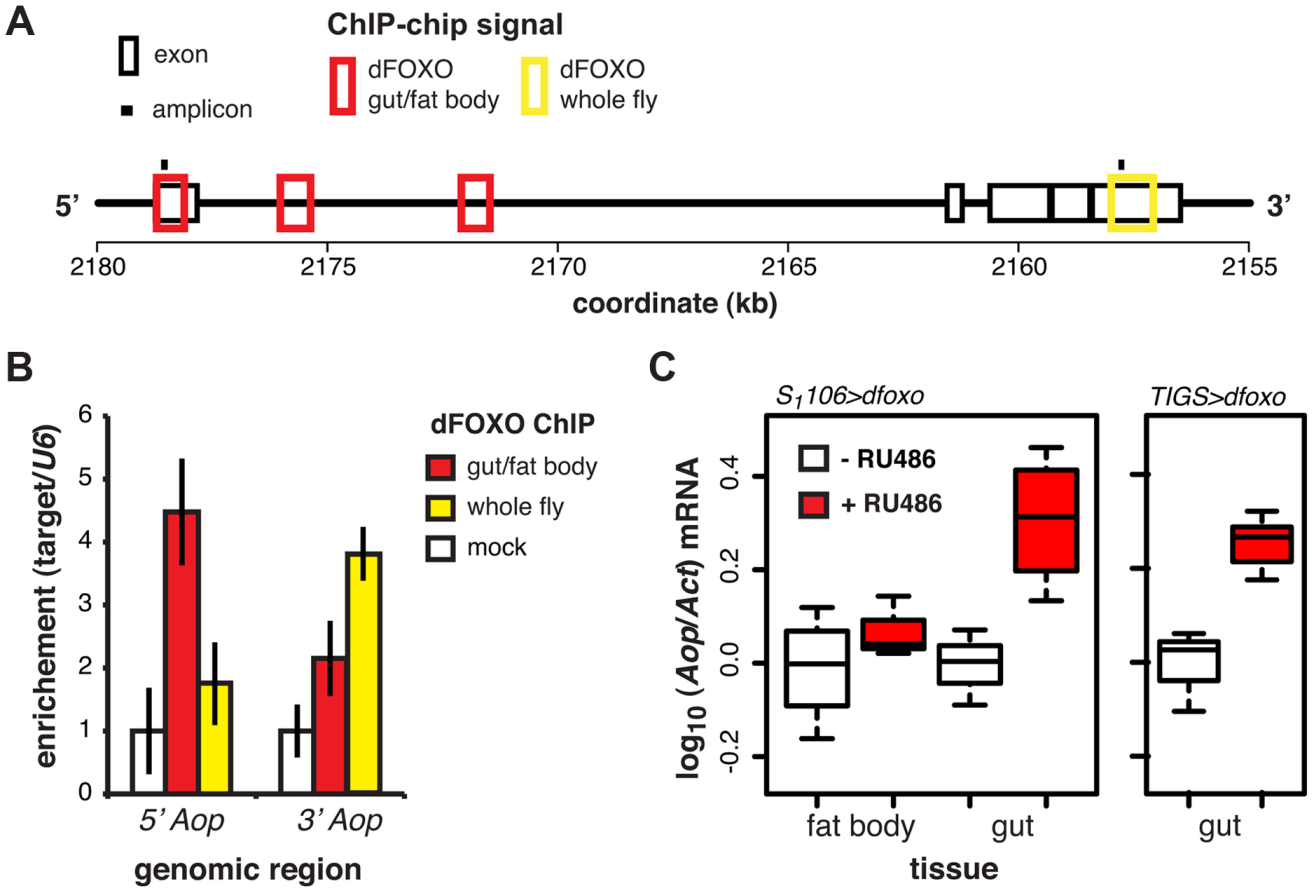
dFOXO regulates expression of *Aop* in the adult gut. **A** Schematic of the *Aop* locus with black boxes representing exons, red boxes – regions detected as bound by GFP-dFOXO in the ChIP-chip experiment on induced *S_1_106>GFP-dfoxo* females (dFOXO gut/fat body), yellow box - region detected as bound by dFOXO in wild-type females (dFOXO whole fly, data obtained from reference [17]), and black bars – position of amplicons used for qPCR in B. **B** The enrichment of 5′ or 3′ end of the *Aop* locus, relative to *U6*, after anti-GFP IP of chromatin from RU486-induced *S_1_106>dfoxo* females (mock), anti-GFP IP of chromatin from RU486-induced *S_1_106>GFP-dfoxo* females (gut/fat body) or anti-dFOXO IP of wild-type female chromatin (whole fly). Means ± SEM of three biological repeats are shown, with enrichment in the mock control set to one. ANOVA on log-transformed data detected significant differences (p = 0.03 per region) and the enrichment of the 5′ region was different in gut/fat body from the mock (one-tailed t-test, p = 6×10^−3^), while the 3′ region was enriched in the whole fly (one-tailed t-test, p = 5×10^−3^). **C** *Aop* mRNA was quantified relative to *Act* by qPCR in guts or fat bodies of *S_1_106>dfoxo*, or *TIGS>dfoxo* flies induced or not with RU486. Boxplots show log-10 derived relative expression with - RU486 values set to zero. Data for *S_1_106>dfoxo* females were analysed with a mixed-effects linear model with dissection batch as a random effect. The effects of RU486, tissue and their interaction was significant (p<0.05) and RU486 caused significant up-regulation of *Aop* in the gut (one-tailed t-test, n = 3–4, p = 2×10^−3^) but not the fat body (one-tailed t-test, n = 4, p>0.05). Significant changes were observed in *TIGS>dfoxo* guts (t-test, n>3, p = 0.02).

The set of direct dFOXO targets included five sequence-specific TFs: *clockwork orange* (*cwo*), *mothers against dpp* (*Mad*), *bunched* (*bun*), *anterior open* (*Aop*) and *CG12054* (summarised in **Figure 2C**). All of these were activated by dFOXO in the gut (**Figure S1F**, for *Aop* see also **Figure 3**). dFOXO also bound its own locus, and inducing the transgenic and intron-less *dfoxo* in *S_1_106>dfoxo* females resulted in an increase in unspliced *dfoxo* (**Figure S1G**), indicating that dFOXO self-activates (**Figure 2C**).

### dFOXO induces an ETS transcriptional repressor in the adult gut

Among the TFs discovered as directly activated by dFOXO in the gut, we were especially interested in *Aop* (a.k.a. *Yan*). This ETS-family transcriptional repressor has a clear human orthologue in the *Etv6* (a.k.a. *Tel*) gene [44]. Aop and *Etv6* display clear conservation of function, with *Aop* involved in tracheal sprouting in flies while *Etv6* is involved in the equivalent process of endothelial sprouting in mammals [45]. Interestingly, mammalian FoxOs and Etv6 act in similar physiological processes: both are tumour suppressors required for maintenance of adult haematopoietic stem cells [30], [46]; indicating that the previously-uncharacterised, functional interplay between the two factors may be evolutionarily conserved. Importantly, *Drosophila* presents a unique opportunity to examine the physiological functions of *Aop*/*Etv6*, because there is no known orthologue in yeast or worm.

ChIP-chip revealed that GFP-dFOXO bound in the promoter-proximal, 5′ end of *Aop*, specifically in the 1^st^ exon and 1^st^ intron (**Figure 3A**). The binding to the 1^st^ exon in the gut/fat body was confirmed with qPCR (**Figure 3B**). Similarly to *dInR*, we previously observed dFOXO bound to the coding region (3′ end) of this gene in whole flies (see [17] and **Figure 3A and B**). The functional significance of this 3′-end binding is unclear [17].

Induction of *dfoxo* in the gut and the fat body resulted in significant induction of *Aop* transcript only in the gut (p = 0.002, **Figure 3C**). To confirm the statistical significance of this tissue-restricted effect of *dfoxo*, we analysed the *Aop* expression data with a mixed-effects linear model and found a significant difference in the way the two tissues respond to RU486 (p<0.05). To confirm that these effects were cell autonomous, we induced *dfoxo* solely in the gut using an RU486-inducible, gut-specific driver, *TIGS* [31]. Feeding RU486 to *TIGS>dfoxo* females also resulted in induction of *Aop* transcript in the gut (**Figure 3C**). Hence, dFOXO induces *Aop* transcription in the gut, most likely through direct binding to the *Aop* promoter in gut cells.

### *Aop* prevents the detrimental co-activation of *dfoxo* and *Pointed*

**Figure 4:**
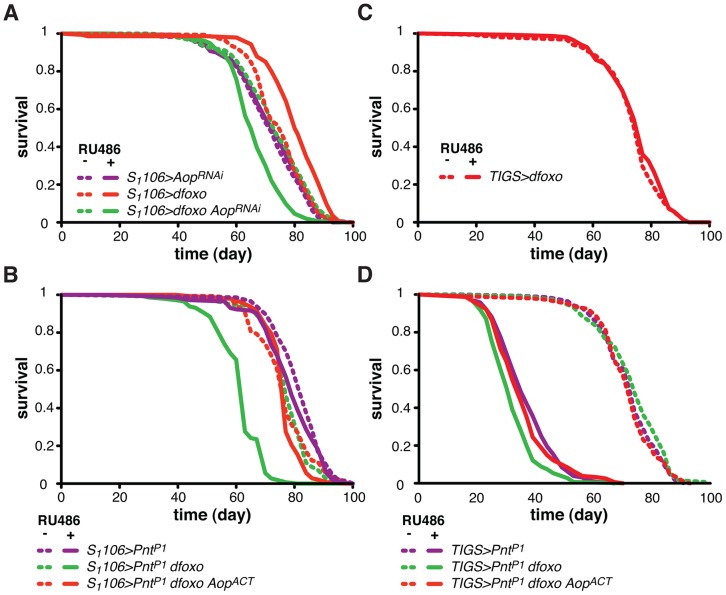
*Aop* prevents the detrimental effects of *dfoxo* and *Pnt* co-activation. **A** Survival of female flies expressing *dfoxo*, *Aop^RNAi^* or both under the control of *S_1_106* in the presence or absence or RU486. Log-rank test revealed significant effect of RU486 for *S_1_106>dfoxo* (p<10^−4^; total dead/censored: − RU486 145/0, + RU486 139/6; median/maximum lifespan: − RU486: 77/87, + RU486 80/90) and *S_1_106>dfoxo Aop^RNAi^* (p<10^−4^; total dead/censored: − RU486 145/2, + RU486 155/2; median/maximum lifespan: − RU486: 74/90, + RU486 65/79) but not *S_1_106>Aop^RNAi^* (p = 0.5; total dead/censored: − RU486 142/0, + RU486 147/4; median/maximum lifespan: − RU486: 72/86, + RU486 74/87). CHP analysis showed that the response to RU486 in *S_1_106>dfoxo Aop^RNAi^* was significantly different from the response in *S_1_106>dfoxo* (p<5×10^−15^) and *S_1_106>Aop^RNAi^* (p<10^−7^) females. **B** Survival of female flies expressing *dfoxo*, *PntP1 and Aop^ACT^* under the control of *S_1_106* in the presence or absence or RU486. Log-rank test revealed significant effect of RU486 for *S_1_106>Pnt^P1^ dfoxo* (p<10^−4^; total dead/censored: − RU486 138/6, + RU486 145/2; median/maximum lifespan: − RU486: 77/90, + RU486 63/83) but not *S_1_106>Pnt^P1^* (p = 0.06; total dead/censored: − RU486 147/0, + RU486 143/2; median/maximum lifespan: − RU486: 84/92, + RU486 79/90) or *S_1_106>Pnt^P1^ dfoxo Aop^ACT^* (p = 0.1; total dead/censored: − RU486 147/3, + RU486 142/3; median/maximum lifespan: − RU486: 77/90, + RU486 77/83). CHP analysis showed that the response to RU486 in *S_1_106>Pnt^P1^* was significantly different from the response in *S_1_106>Pnt^P1^ dfoxo* (p<10^−15^) but not in *S_1_106>Pnt^P1^ dfoxo Aop^ACT^* (p = 0.5) females. **C** Survival of *TIGS>dfoxo* female flies in the presence or absence of RU486. Log-rank test detected no significant differences (p = 0.3; total dead/censored: − RU486 152/1, + RU486 149/2; median/maximum lifespan: − RU486: 77/87, + RU486 77/86). **D** Survival of female flies expressing *dfoxo*, *Pnt^P1^ and Aop^ACT^* under the control of *TIGS* in the presence or absence or RU486. Log-rank test revealed significant effect of RU486 for *TIGS>Pnt^P1^* (p<10^−4^; total dead/censored: − RU486 140/2, + RU486 136/7; median/maximum lifespan: − RU486: 74/86, + RU486 35/50), *TIGS>Pnt^P1^ dfoxo* (p<10^−4^; total dead/censored: − RU486 146/3, + RU486 148/1; median/maximum lifespan: − RU486: 74/85, + RU486 32/45) and *TIGS>Pnt^P1^ dfoxo Aop^ACT^* (p = 0.1; total dead/censored: − RU486 143/1, + RU486 155/1; median/maximum lifespan: − RU486: 72/85, + RU486 35/55). CHP analysis showed that the response to RU486 in *TIGS>Pnt^P1^* was significantly different from the response in *TIGS>Pnt^P1^ dfoxo* (p = 2×10^−4^) but not in *TIGS>Pnt^P1^ dfoxo Aop^ACT^* (p = 0.8) females.

To understand the relationship between *dfoxo* and its target TF, *Aop*, we next examined the physiological role played by *Aop* in the context of tissue-restricted *dfoxo* induction and lifespan. Since *Aop* is an essential gene [47], [48], we chose to knock it down in the adult gut and fat body using a short-hairpin RNAi construct with no predicted off-targets [49]. Driving this construct with a ubiquitous, constitutive driver (*daughtelessGAL4*) resulted in the expected embryonic lethality. Inducing this construct in the adult gut and fat body, using the *S_1_106* driver, reduced the levels of *Aop* mRNA by ∼70% (p = 0.04, **Figure S2A**) but had no major effect on lifespan (**Figure 4A**), revealing that, in a wild-type fly, *Aop* in these tissues is not limiting for survival.

To test if *Aop* is required for the *dfoxo*-induced longevity, we simultaneously expressed *dfoxo* and knocked-down *Aop*. Surprisingly, while *dfoxo* alone extended lifespan, this combined treatment was detrimental to the fly (**Figure 4A**), indicating that *Aop* is required to prevent some toxic effect of *dfoxo* activation [39]. To determine the statistical significance of this synergistic effect, we used Cox Proportional Hazards (CPH) analysis [50]. This type of survival analysis can determine the significance of individual covariates as well as their interaction. We used “genotype” and “RU486” as individual covariates and found that the response to RU486 was significantly different in *S_1_106>dfoxo Aop^RNAi^* genotype from the response in either *S_1_106>dfoxo* or *S_1_106> Aop^RNAi^* genotypes (interaction of “genotype” and “RU486”, p = 5×10^−15^ and p = 1×10^−7^, respectively). This confirmed the synthetic interaction between the loss of *Aop* and induction of *dfoxo*. Importantly, expressing a control RNAi construct targeting *GFP* with *S_1_106* driver did not prevent *dfoxo* from extending lifespan (**Figure S2B**).

We next examined the mechanism underlying this toxic effect of inducing *dfoxo* in the absence of *Aop*. *Aop*'s activity is known to counteract that of *Pointed* (*Pnt*), an ETS-family transcriptional activator [51], [52]. These two TFs regulate the same genes through binding to the same regulatory elements but with opposing outcomes [51]–[53]. Hence, the synthetic toxicity of *Aop* loss- and *dfoxo* gain-of-function indicated that co-activation of *Pnt* and *dfoxo* could be highly detrimental. To test this, we induced the constitutively active form of *Pnt* (*Pnt^P1^*) [51] in the adult gut and fat body. This resulted in a marginal negative effect on lifespan (p = 0.06) and, similarly to the phenotype of *Aop* loss and *dfoxo* gain, the co-induction of *dfoxo* and *Pnt^P1^* was highly detrimental (**Figure 4B**).

It was possible that dFOXO induced the transcription of *Aop* in order to avert co-activation of PNT and the resulting detrimental synergistic effect. Indeed, the detrimental effect of combined dFOXO and PNT activity was completely rescued by additional activation of AOP, achieved by expression of a constitutively active form of *Aop* [48], called *Aop^ACT^*, in addition to *dfoxo* and *Pnt^P1^* (**Figure 4B**). The significance of this effect was confirmed by CPH analysis, which showed that the response to RU486 in *S_1_106>Pnt^P1^* genotype was significantly different from that in *S_1_106>Pnt^P1^ dfoxo* genotype (p<10^−15^), but not from the response observed in *S_1_106>Pnt^P1^ dfoxo Aop^ACT^* (p = 0.5). Importantly, induction of *Aop^ACT^* did not interfere with the induction of *dfoxo* or *Pnt^P1^* in the *S_1_106>Pnt^P1^ dfoxo Aop^ACT^* females compared to the *S_1_106>Pnt^P1^ dfoxo* females (**Figure S2C**).

dFOXO regulates *Aop* transcription specifically in the gut (**Figure 3C**). For this reason we next examined whether the genetic interactions we observed between *dfoxo* and *Aop*/*Pnt* could be recapitulated with the gut-specific *TIGS* driver. This driver has been successfully used by others to determine the contribution of the gut to *S_1_106*-triggered phenotypes [54]. The gut is a highly regionalised organ with several cell types [55], [56] and we confirmed that the two drivers activate transgene expression in similar regions (**Figure S3A**), both driving expression in the enterocytes (**Figure S3B**). Feeding RU486 to *TIGS>dfoxo* females had no significant effect on lifespan (**Figure 4C**), even though *dfoxo* was induced in the gut (**Figure S1A**) and activated the expression of *Aop* (**Figure 3C**). With the caveat that the levels of expression achieved with *TIGS* and *S_1_106* in the gut are not identical (**Figure S1A**, **S3A**) and the two drivers may not drive in completely overlapping subsets of gut cells (**Figure S3A** and **B**), our data indicate that activation of dFOXO in the gut alone is insufficient for lifespan extension.

On the other hand, the induction of *Pnt^P1^* solely in the adult gut was highly detrimental for the fly (**Figure 4D**), while RU486 had no effect on the lifespan of the *TIGS*-alone or *UAS-Pnt^P1^*-alone controls (**Figure S3C** and **D**). The interaction between *dfoxo* and *Pnt* revealed with the *TIGS* driver was similar to that observed with *S_1_106*: induction of *dfoxo* exacerbated the toxicity of *Pnt^P1^* and this, in turn, could be remedied by further induction of *Aop^ACT^* (**Figure 4D**). While the magnitude of this synergistic effect was smaller with *TIGS* than *S_1_106*, CHP analysis confirmed that the response to RU486 in *TIGS>Pnt^P1^* females was significantly different from that in *TIGS>Pnt^P1^ dfoxo* (p<10^−4^), but not in *TIGS>Pnt^P1^ dfoxo Aop^ACT^* females (p = 0.8). Hence, the dFOXO-PNT-AOP interaction can be mapped specifically to the gut. Note, however, that we cannot exclude the possibility of a similar interaction also occurring in the fat body.

Overall, our data are consistent with dFOXO up-regulating *Aop* transcription in the gut in order to counteract the activity of PNT. In this way, dFOXO prevents the detrimental effect that would result from dFOXO being active at the same time as PNT in that organ.

### dFOXO and AOP share binding locations

**Figure 5:**
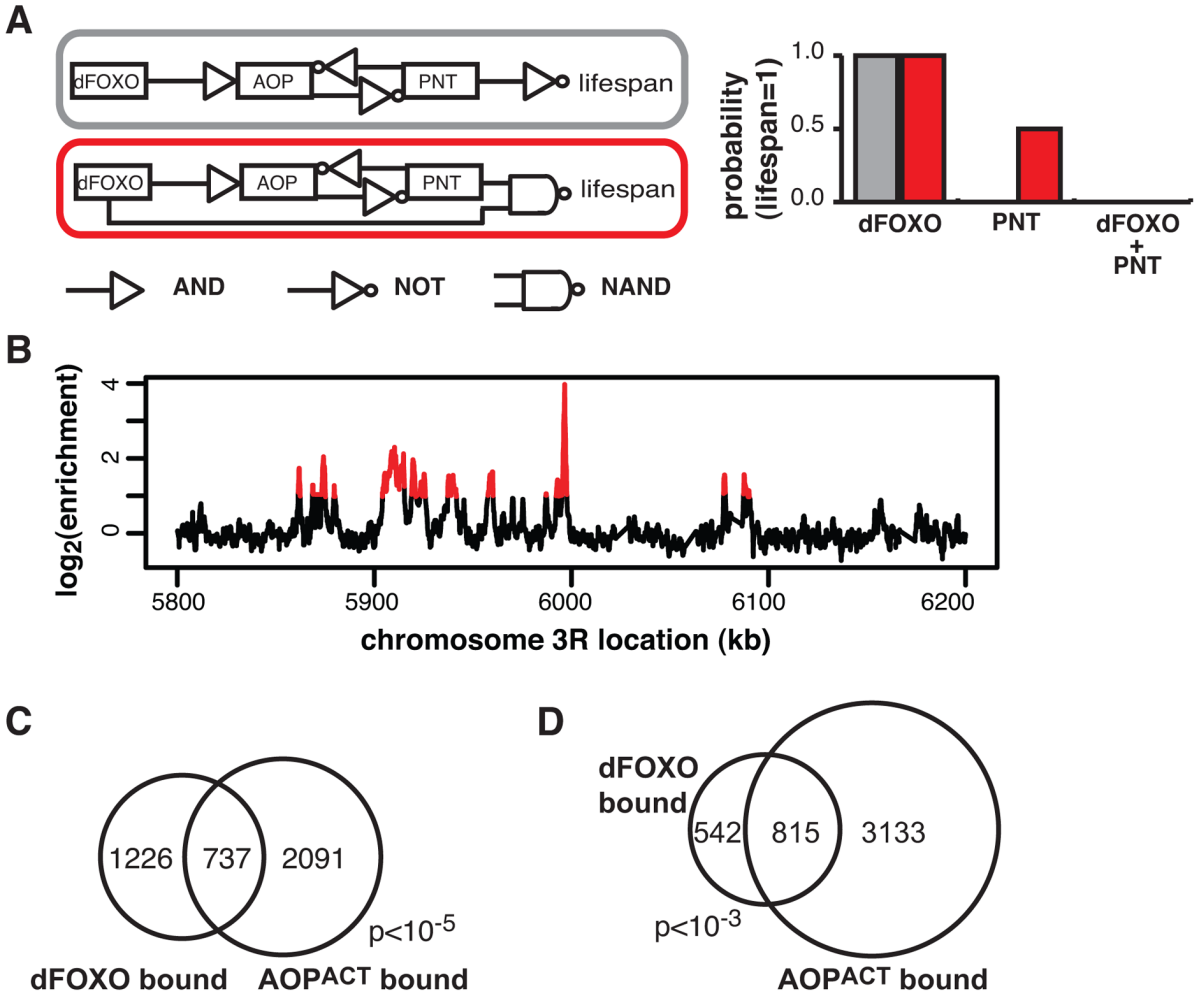
dFOXO and AOP^ACT^ share binding sites across the genome. **A** Boolean network models of dFOXO-AOP-PNT-lifespan interaction. The linear model is indicated in grey and the feedforward model in red. The logic gate symbols for AND, NOT and NAND (NOT AND) are used, and in the case shown NAND means that only the combined effect of dFOXO and PNT is detrimental to lifespan. For each model the probability of a positive outcome for lifespan (lifespan = 1; y axis) after 1000 state transition was determined using Markov chain simulations after fixing either dFOXO or PNT or both as active ( = 1; x axis). Only the feedforward model can capture the synergistic negative effects of the two TFs. **B** ChIP-chip traces, showing the enrichment (log_2_-transformed) of the FLAG-AOP^ACT^-immunoprecipitated DNA over total chromatin, are averages of three biological repeats after subtraction of the mock control and are shown over a region of chromosome 3R. Red denotes the enrichment associated with peak regions. **C** Proportional Venn diagram showing the overlap between the genes in the vicinity (<1 kb) of GFP-dFOXO or FLAG-AOP^ACT^ binding in the gut/fat body (Hypergeometric test, p<10^−5^). **D** Proportional Venn diagram showing the overlap in GFP-dFOXO-bound and FLAG-AOP^ACT^-bound genomic sites. Note that the average overlap is shown, since one peak in one set can overlap multiple peaks in the other due to differing peak lengths: 819 GFP-dFOXO-bound and 810 FLAG-AOP^ACT^-bound sites are in the overlap. Bootstrap analysis revealed the overlap as significant (p<10^−3^).

Intuitively, the genetic interactions observed between *dfoxo* and *Aop*/*Pnt* were not consistent with a simple, linear cascade of dFOXO acting on AOP/PNT to influence lifespan (**Figure 5A**). To formally examine this, we used Boolean network modelling. We created network configurations with dFOXO, AOP, PNT and “lifespan” as nodes that can take values of 0 or 1 (inactive/short life or active/long life) and described the relationships between them using Boolean logic operators. We then perturbed the network by fixing either dFOXO or PNT or both as “active” and examined the probability that lifespan will take on the value of 1 (long life) after 1000 state transition using Markov chain simulations. This type of modelling formalised what was intuitively evident: the synergistic negative effect of *dfoxo* and *Pnt* co-activation could be recapitulated by a network circuit that includes a feed-forward loop between *dfoxo* and *Pnt* with a negative effect on lifespan (described by a NAND operator), but not by a linear cascade (**Figure 5A**).

Feed-forward loops involve co-regulation of the same targets, and are often accompanied by extensive overlaps in genomic sites bound by the TFs [57]. To gather further evidence for the existence of this feed-forward loop, we determined the relationship between the sites bound by the AOP/PNT couple and dFOXO. We performed ChIP-chip on flies expressing FLAG-AOP^ACT^, since both PNT and AOP are known to bind the same sequences, competing for the same sites [51]–[53].

To facilitate the comparisons with dFOXO, we used the *S_1_106* driver and the RU486 inducer, with the control IP performed with an anti-FLAG antibody on chromatin from flies expressing untagged AOP^ACT^ (ArrayExpress accession number: E-MTAB-1306). We discovered ∼4000 genomic regions bound by FLAG-AOP^ACT^ neighbouring some 3000 genes, including the *dfoxo* locus, with good correlation between biological repeats (**Figure S4A**). An example of peaks is given in **Figure 5B**. The binding locations were associated with genes and tended to occur in the 5′ region (**Figure S4B**), consistent with a role in promoter-proximal regulation of transcription. Similarly to the recently published AOP binding locations observed in larvae [58], we found that AOP tended to bind long stretches of DNA, longer than dFOXO (**Figure S4C**). At the same time, over 80% of the regions bound in adult gut/fat body were distinct from those bound in larvae (**Figure S4D**).

We found a significant and substantial overlap in the genes bound by GFP-dFOXO and those bound by FLAG-AOP^ACT^ (p<10^−5^, **Figure 5C**), indicating that AOP/PNT and dFOXO may regulate numerous genes in common. Furthermore, dFOXO did not just bind in the vicinity of the same genes but actually to the same regions of the DNA: 60% of GFP-dFOXO-bound regions directly overlapped regions of FLAG-AOP^ACT^ binding (**Figure 5D**). This striking and significant (p<10^−3^) overlap in sites bound corroborates the existence of a feed-forward loop between AOP/PNT and dFOXO. Furthermore, the genetic interactions we observed between *dfoxo* and *Pnt*/*Aop* are likely to be mediated by functional interactions on the shared regulatory regions.

### *Pnt* and *dfoxo* synergistically affect lipid metabolism

**Figure 6:**
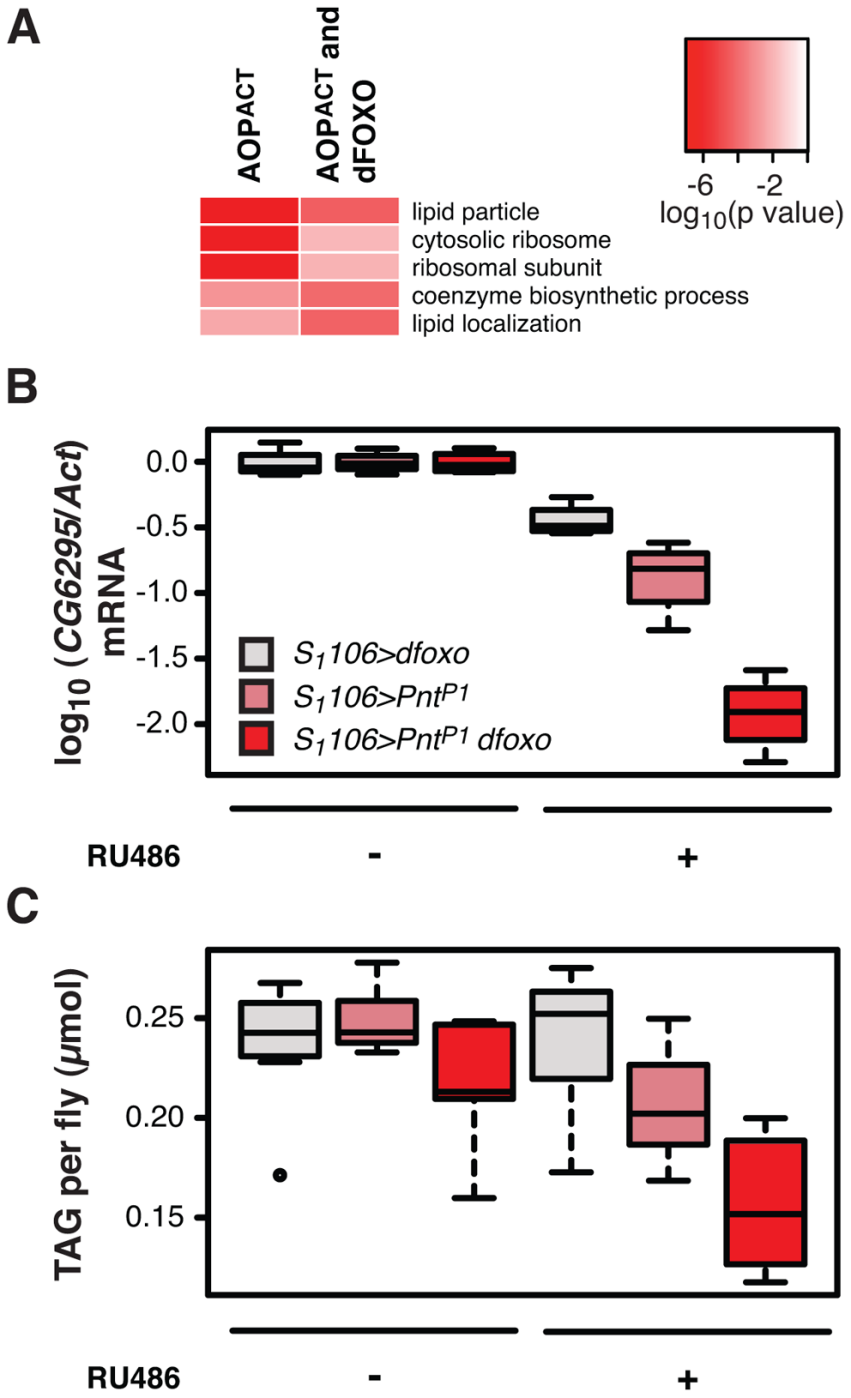
*dfoxo* and *Pnt* act synergistically on lipid metabolism. **A** Top three most significantly enriched GO categories within genes bound by FLAG-AOP^ACT^ alone or FLAG-AOP^ACT^ and dFOXO, as determined by EASE analysis. The intensity of red indicates log-10 derived p value associated with the enrichment. The complete GO analysis is given in **Dataset S1**. **B** *CG6296* mRNA was quantified relative to *Act* by qPCR in the females of the indicated genotypes, induced or not with RU486. Boxplots show log-10 derived relative expression with − RU486 values set to zero. Data were analysed with a linear model and the effects of RU486, genotype and their interaction were significant (n = 4, p<10^−4^), with *S_1_106>dfoxo Pnt^P1^* + RU486 condition being different to all others (t-test , p<10^−4^). **C** The levels of TAG were quantified under the same conditions as in B. Data were analysed with a linear model and the effects of RU486 (p = 2×10^−4^), genotype (p<10^−4^) and their interaction (p = 0.01) were significant (n = 8, where 10 measurements were made and the lowest and highest measurement removed from each group). The *S_1_106>dfoxo Pnt^P1^* + RU486 condition was different from all others (t-test , p<0.05). The genotypes are denoted with the same colours in B and C, with the legend given in B.

The strength of the negative synergistic effect observed between *dfoxo* and *Pnt* prompted us to seek out its physiological basis. To initiate this investigation, we looked at the GO categories that are over-represented in genes bound by FLAG-AOP^ACT^. The most over-represented functional category was “lipid particle” (p = 3×10^−17^, **Figure 6A**), a GO category that includes lipid droplets, sites of cellular lipid storage. This was also the most over-represented category in the set of genes bound by both FLAG-AOP^ACT^ and GFP-dFOXO (p = 3×10^−5^, **Figure 6A**), indicating that PNT/AOP and dFOXO together regulate lipid metabolism.

Activation of dFOXO in the aging gut represses two gastric lipases, encoded by *lipA* (a.k.a. *magro*) and *CG6295* genes [39]. These two lipases are thought to facilitate assimilation of ingested lipids, and their repression by dFOXO results in a decrease in lipid stores and a shortened lifespan [39]. We observed the detrimental effects of dFOXO activation in the gut to be dependent on simultaneous activation of PNT (**Figure 4B, C and D**), prompting us to examine whether the expression of either *lipA* or *CG6295* was synergistically regulated by *dfoxo* and *Pnt*. We used the *S_1_106* driver, where the synergistic effect on lifespan is pronounced, and found that both *dfoxo* and *Pnt^P1^* resulted in repression of *CG6295*, 3-fold and 8-fold, respectively (**Figure 6B**). When the two TF were co-expressed, the reduction in *CG6295* was even greater, reaching 80-fold reduction after only 5 days of transgene induction (**Figure 6B**). Indeed, a linear model revealed a significant difference in the way the three genotypes responded to RU486 (p<10^−4^), and the amount of *CG6295* transcript was significantly different between *S_1_106*>Pnt^P1^ + RU486 or *S_1_106>dfoxo* + RU486 and *S_1_106*>*Pnt^P1^ dfoxo* + RU486 conditions (p<10^−4^). Hence *dfoxo* and *Pnt* synergistically repress the CG6295 lipase. The effect of the two TFs on *lipA* was similar but smaller, and there was no significant difference between *S_1_106>dfoxo* + RU486 and *S_1_106*>*Pnt^P1^ dfoxo* + RU486 conditions (p>0.05, **Figure S5A**). Note that neither AOP^ACT^ nor dFOXO bind in the vicinity of *CG6295* and hence the effects on the expression of this gene are likely to involve an intermediate factor.

To examine whether the synergistic effect on the expression of the *CG6295* lipase had a significant physiological consequence, we determined the levels of triacylglycerol (TAG) stores in flies expressing *dfoxo*, *Pnt* or both, under the control of the *S_1_106* driver. We found that the levels of TAG paralleled the levels of *CG6295* transcript (**Figure 6C**) and, using a linear model, we confirmed that the effects of RU486 on the TAG levels in the 3 genotypes were significantly different (p = 0.01), with the TAG being significantly more depleted in *S_1_106>PntP1 dfoxo* flies than in the other two genotypes after only 5 days of RU486 feeding (p<0.02, **Figure 6C**).

The effect on *CG6295* expression and TAG levels could be symptomatic of a more general loss of gut integrity. The function of the gut as a barrier is a good surrogate for overall gut integrity and is important for survival [59]. The effectiveness of this barrier can be assessed by feeding flies a food containing a dye (fluoresceine) and scoring the number of flies in which the gut is unable to exclude this dye from the whole body (“smurf” phenotype) [59]. We found that driving *dfoxo* and *Pnt^P1^* for 3 weeks using *S_1_106* did not increase the proportion of smurfs in the population (**Figure S5B**), while the effect on lipid metabolism was evident after only 5 days (**Figure 6C**). This confirmed that the effect on lipid metabolism was not due to a general loss of gut integrity. In addition, we also observed no significant changes in feeding behaviour of *S_1_106*>*Pnt^P1^ dfoxo* females after RU486 treatment, as assessed with the proboscis-extension assay (**Figure S5C**).

Based on these data, we propose that the detrimental synergy between *dfoxo* and *Pnt* arises from a mis-regulation of lipid metabolism resulting in a profound drop in TAG stores. Indeed, *S_1_106>PntP1 dfoxo* flies were starvation sensitive after 5 days of RU486 feeding, and this sensitivity could be reversed by co-induction of *Aop^ACT^* (**Figure S5D**).

### Activation of AOP alone can extend lifespan

**Figure 7:**
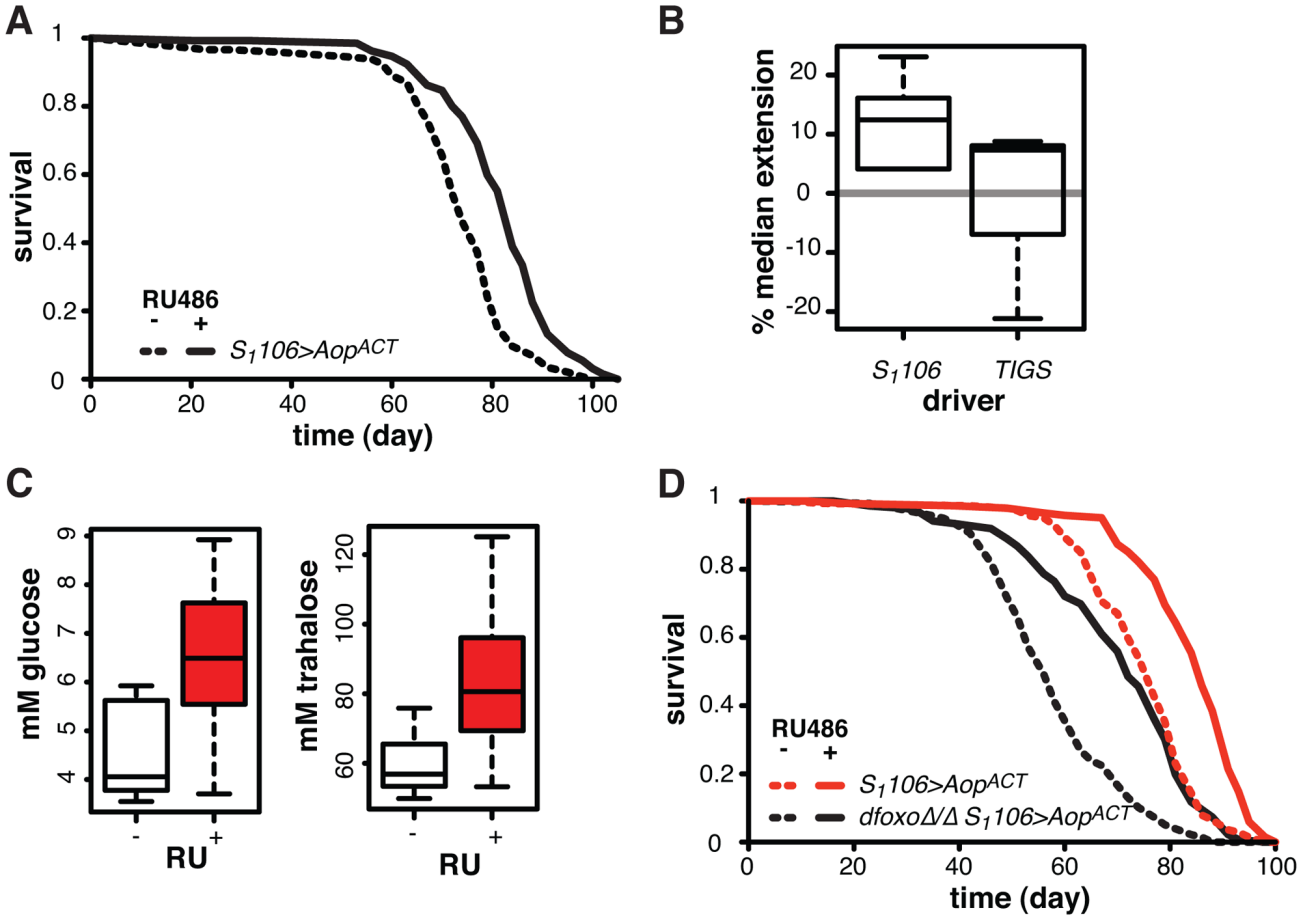
*Aop* extends lifespan. **A** Survival of *S_1_106>Aop^ACT^* female flies in the presence or absence or RU486. Log-rank test detected significant differences (p = 2×10^−10^; total dead/censored: − RU486 145/1, + RU486 129/3; median/maximum lifespan: − RU486: 74/87, + RU486 84/97). **B** Median lifespan extension achieved by RU486 feeding in *S_1_106>Aop^ACT^* (6 experiments) or *TIGS>Aop^ACT^* (3 experiments) females. Log-rank test detected significant extension (p<0.05) of lifespan in 6 out of 6 *S_1_106>Aop^ACT^* and in 1 out of 3 *TIGS>Aop^ACT^* trials. In one *TIGS>Aop^ACT^* trial lifespan was shortened. **C** Haemolymph glucose and trehalose in *S_1_106>Aop^ACT^* females fed or not RU486, where RU486 had a significant effect in each case (t-test, p = 0.01 and 0.02 respectively; n = 8 where 10 measurements were made and the highest and lowest measurement removed from each group). **D** Survival of *S_1_106>Aop^ACT^* or dfoxoΔ/Δ *S_1_106>Aop^ACT^* female flies in the presence or absence or RU486. Log-rank test detected significant differences for both: *S_1_106>Aop^ACT^* (p<10^−4^; total dead/censored: − RU486 139/3, + RU486 136/7; median/maximum lifespan: − RU486: 77/87, + RU486 86/94) and dfoxoΔ/Δ *S_1_106>Aop^ACT^* (p<10^−4^; total dead/censored: − RU486 138/0, + RU486 136/4; median/maximum lifespan: − RU486: 56/81, + RU486 72/90). CHP analysis revealed significant effects of RU486 (p<10^−15^) and *dfoxo* (p<10^−15^) but no significant difference in the response to RU486 between the two lines (p = 0.2).

We next focused on other possible roles of *Aop* in *Drosophila* lifespan. Expression of the activated form of *Aop* in the flies co-expressing *dfoxo* and *Pnt^P1^* with *S_1_106* driver had a substantially beneficial effect on lifespan (**Figure 4B**). This was due, in part, to its role in preventing dFOXO and PNT co-activation, but we hypothesised that activation of AOP might additionally increase the lifespan of wild type flies. We induced expression of the activated form of AOP (*Aop^ACT^*) using the *S_1_106* driver and the RU486 inducer in an otherwise wild-type adult female. This resulted in significant lifespan-extension (p = 2×10^−10^), increasing the median by 14% and maximal lifespan by 11% (**Figure 7A**), while RU486 feeding had no effect on the lifespans of the driver- or transgene-alone controls (**Figure S6A** and **B**). This effect was robust and observed in six independent experiments, performed in the course of three years, with average median lifespan extension of 12% (**Figure 7B**). Furthermore, independently generated, FLAG-tagged *Aop^ACT^* also extended lifespan (**Figure S6C**).

To examine whether this lifespan-extending effect could be localised to the same tissues where the interaction between *dfoxo* and *Pnt*/*Aop* occurs, we drove *Aop^ACT^* with the gut specific *TIGS* driver. Induction of *Aop^ACT^* using the TIGS driver did not extend lifespan (**Figure 7B**), despite transgene induction in the gut with both *TIGS* and *S_1_106* drivers (**Figure S6D**). Although the induction levels were lower with *TIGS* (**Figure S6D**), they must still be physiologically relevant because, even with this driver, *Aop^ACT^* could partially remedy the toxicity of *Pnt* and *dfoxo* co-activation (**Figure 4D**).

Our data are consistent with two roles of *Aop* in lifespan modulation: (1) *Aop* can counteract the negative effect of *dfoxo* and *Pnt* co-activation in the gut, (2) its additional activation in the fat body extends wild-type lifespan. The lifespan extension by *Aop^ACT^* in flies co-expressing *dfoxo* and *Pnt^P1^* under *S_1_106* control most likely combines the two beneficial effects of *Aop* activation, since the effect appears greater in that context than in the otherwise wild-type female (median increased by 22% between *S_1_106>dfoxo Pnt^P1^* + RU486 and *S_1_106>dfoxo Pnt^P1^ Aop^ACT^* + RU486, **Figure 4B**, versus 12% average extension in wild-type, **Figure 7B**).

We examined other phenotypes triggered in *S_1_106>Aop^ACT^* females by RU486 feeding, focusing on phenotypes often associated with lifespan extension. RU486 feeding did not cause any significant changes in starvation, hydrogen peroxide or DDT resistance; any changes to whole body trehalose, glycogen or lipid content; feeding or fecundity (**Figure S7**). However, we did find that induction of *Aop^ACT^* in the adult gut and fat body resulted in increased circulating sugars (**Figure 7C**), a diabetic phenotype potentially indicative of slightly reduced IIS. *Pnt* is known to promote IIS in larvae and its gain-of-function stimulates clearance of circulating sugars [60], consistent with the converse phenotype we observed in *S_1_106>Aop^ACT^* adults.

Since FoxOs are inhibited by IIS [61], *Aop* could act to reduce IIS and activate dFOXO, thereby increasing the lifespan of otherwise wild-type females. However, *Aop^ACT^* extended lifespan in complete absence of *dfoxo* (**Figure 7D**), and CPH analysis found no evidence for a difference in the response to RU486 in *dfoxo*Δ/Δ *S_1_106>Aop^ACT^* versus *S_1_106>Aop^ACT^* females (p = 0.2). Hence, *Aop* extends wild-type lifespan independently of *dfoxo*.

### dFOXO and AOP share targets in the fat body

**Figure 8:**
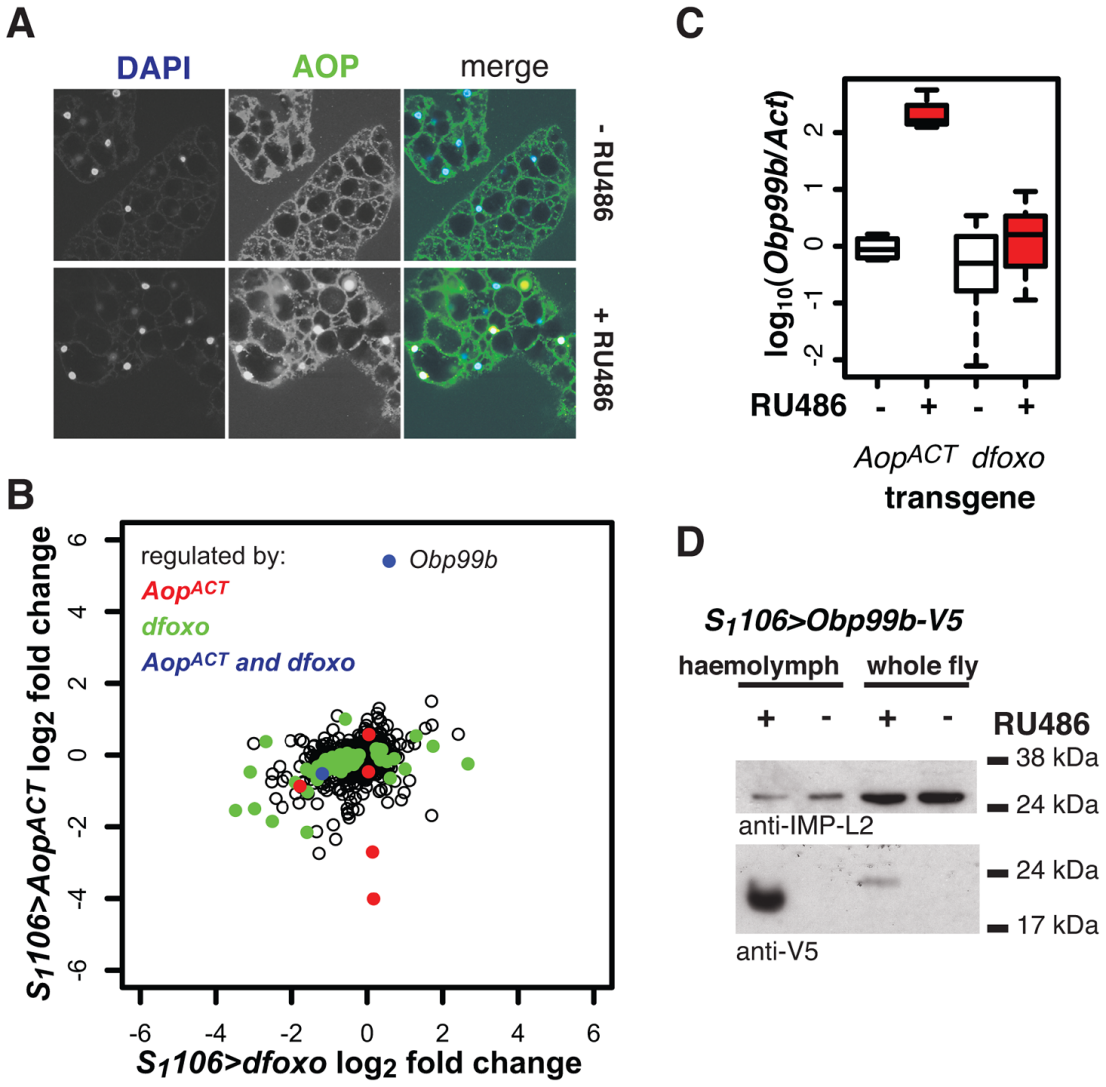
*Aop* and *dfoxo* both regulate a humoral factor, Obp99b, in the fat body. **A** AOP was visualised by immunofuorescence in fat bodies of *S_1_106>Aop^ACT^* flies induced or not with RU486. In the merged image, AOP is shown in green, DAPI-stained nuclei in blue. AOP accumulates in nuclei of 26±6% of fat body cells. **B** Genes regulated by RU486 induction in fat bodies of *S_1_106>Aop^ACT^* flies were determined by microarray analysis and compared to changes in the fat body upon induction of *S_1_106>dfoxo*. Mean log_2_ fold change caused by induction of *Aop^ACT^* (y-axis) is plotted against that caused by induction of *dfoxo* (x-axis). Genes with significant differential expression (at 20% FDR) upon induction of *Aop^ACT^* are shown in red, *dfoxo* in green and both *Aop^ACT^* and *dfoxo* in blue. The location of the *Obp99b* gene on the graph is indicated. **C** The levels of *Obp99b* mRNA in abdominal fat body mRNAs were determined relative to *actin* by qPCR upon RU486 induction in *S_1_106>Aop^ACT^* (n = 4) or *S_1_106>dfoxo* (n = 8) female flies. Boxplots show log-10 derived ratios scaled to set the −RU486 condition to 0. Data were analysed using a mixed-effects linear model with genotype and RU486 as main effects and dissection batch as a random effect. Both main effects as well as their interaction were significant (p<0.01), and one-tailed t-test indicated the +RU486 condition had more Obp99b transcript for each genotype (p<0.05). **D** Obp99b-V5 was detected in haemolymph (1 µl) of *S_1_106>Obp99b-V5* female flies, fed or not RU486, or in whole-fly extracts (equivalent of half a fly) using anti-V5 antibody. The secreted IMP-L2 protein was used as a loading control.

We further examined the function of *Aop* in the fat body, where its activity appears important for lifespan but where it is not regulated by *dfoxo*. We found that AOP transcript and protein levels were strongly induced in the fat body when *S_1_106>Aop^ACT^* females were fed RU486 (**Figure S8A**), but in only a portion (∼26±6%) of the fat body cells, suggesting heterogeneity of the tissue (**Figure 8A**, for quantification see **Figure S8B**). This mosaic expression was not due to the driver, because *S_1_106* drove GFP expression in all the cells of the tissue (**Figure S8C**).

To elucidate the function of *Aop* in the fat body we used microarray analysis on dissected tissue to identify changes in gene expression upon RU486 feeding in *S_1_106>Aop^ACT^* females (ArrayExpress accession number: E-MTAB-927). The induction of AOP^ACT^ in only a few cells of the fat body precluded identification of many genes with significant changes upon RU486 feeding. We identified 8 genes (p value cut off of 0.00017, corresponding to 20% FDR, **Figure 8B**). Two of these were also regulated by *dfoxo* (**Figure 8B**), and this modest overlap was statistically significant (p = 2×10^−3^). The functions regulated by the two factors, as determined by Catmap analysis (**Figure S9A**) were also partially overlapping. For example, both *dfoxo* and *Aop^ACT^* had a negative effect on respiratory electron transport chain (p = 10^−21^ and p = 7×10^−9^ respectively), while *Aop^ACT^* alone had an effect on carbohydrate metabolism (p = 9×10^−18^ for *Aop^ACT^*, p>0.05 for *dfoxo*) (**Figure S9A**). Interestingly, we found that the lifespan benefits from the induction of *Aop^ACT^*, *dfoxo* or both were not significantly different (**Figure S9B**). This indicated that the effects of the two factors are not additive and, in turn, that the two are likely to affect lifespan via the same or related physiological processes, in an otherwise wild-type background.

Interestingly, the most strongly up-regulated gene in the fat body upon *Aop^ACT^* induction was also up-regulated by dFOXO in the same tissue, albeit less strongly (**Figure 8B**). These observations were confirmed by qPCR (**Figure 8C**). This gene encodes a signal peptide followed by an odorant binding protein (Obp) domain - *Obp99b*. Non-sensory Obps are thought to bind and carry lipophilic compounds, fulfilling similar functions to the mammalian lipocalin family proteins, rather than acting in odour perception [62], [63]. Obp99b could represent a humoral factor, induced by both *dfoxo* and *Aop*, mediating inter-tissue communication. Such inter-tissue communication is known to be important in the physiological effects of tissue-restricted *dfoxo* induction [4]. To establish that this Obp was actually secreted *in vivo* we expressed a transgene encoding a V5-tagged Obp99b with the *S_1_106* driver. Feeding RU486 to these flies confirmed the predicted enrichment of Obp99b-V5 in the haemolymph (**Figure 8D**) and revealed that its transcriptional up-regulation is enough to release it in circulation. Hence, in the fat body, *Aop^ACT^* and *dfoxo* regulate a common humoral factor, whose function warrants further investigation.

## Discussion

Our study clearly demonstrates the *in-vivo* complexity of interactions that occur between FoxOs and the TFs they regulate. It emphasises the need to untangle the tissue-specific transcriptional networks within which FoxOs act in order to understand the role of FoxO factors in lifespan.

We show that dFOXO activates the transcription of an ETS repressor, *Aop*, in adult *Drosophila* gut to antagonise an ETS activator, *Pnt*, and avert the detrimental effects of the co-activation of dFOXO and PNT. This interaction most likely takes place at the gene promoters/enhances where AOP may directly displace PNT from a dFOXO bound region. Whether this interaction is facilitated by a direct protein-protein interaction between dFOXO and AOP, or by juxtaposition of dFOXO and AOP binding sites, remains to be determined.

Others have shown that induction of *dfoxo* solely in the gut is detrimental for lifespan [39]. In our outbred, wild-caught background, this detrimental effect is conditional on co-activation of *Pnt*. Despite these differences, the deleterious physiological outcome is likely to be based on mis-regulation of lipid metabolism genes and a drop in TAG stores in both cases. In parallel, the activation of AOP in the gut/fat body of otherwise wild-type females has only two phenotypes: increase in lifespan and increase in circulating sugars. The increase in circulating sugars is a metabolic phenotype, generally not detrimental to fly lifespan as it can be observed in a number of long-lived IIS mutants [64], [65]. Together with the effects observed upon *dfoxo*/*Pnt* co-activation, it indicates that the *Aop*/*Pnt* couple have metabolic functions in the adult fly.

FoxOs are regulated by AKT [61], while ETS factors, such as AOP and PNT, are regulated by ERK [51]. In turn, both ERK and AKT are activated in response to activation of receptor tyrosine kinases (RTK) [66]. Viewed in this context, the interaction between dFOXO, inhibited by AKT, and AOP, inhibited by ERK, insures that the two branches down-stream of RTKs are coordinated. Our findings reveal the mis-coordination of the two branches to be highly detrimental, with the cross-talk between *dfoxo* and *Aop* set up to prevent it. This mechanism for coordination of the activities of the two branches, via interactions between FoxO and ETS factors, is potentially relevant in numerous contexts beyond *Drosophila* lifespan.

Dissecting the relationship between dFOXO and AOP led us to the discovery of a second beneficial role for AOP and the identification of *Aop* as a longevity determinant in its own right. *Aop* has an important and well-established role in *Drosophila* development. The gene was initially isolated as encoding a negative regulator of neural development, in the context of the *Drosphila* eye [47]. Subsequent studies revealed it to be a general inhibitor of differentiation [48], [67]. Its role in the adult has not been investigated and its effect on lifespan was completely unsuspected.

Several contingent observations lead us to speculate that the role of *Aop* as a fly longevity gene, as well as its interaction with *dfoxo*, will be conserved in its mammal orthologue *Etv6*: Aop and *Etv6* display clear conservation of function in other physiological processes [45]; the common roles of *dfoxo* and *Aop* in fly lifespan are paralleled by shared roles of FoxO factors and *Etv6* in mammals [30], [46]; the neuroendocrine axes controlling growth are important in mammalian lifespan [68] and *Etv6* has been identified as associated with human height in genome-wide association studies [69], [70].

## Materials and Methods

### Fly lines, husbandry, lifespan and physiological assays

*S_1_106* [1], [31], *TIGS* [31] *UAS-dfoxo* [1], *UAS-GFP-dfoxo* [32], *UAS-Aop^ACT^* [48], *UAS-Obp99b-V5.5* [71], *UAS-Pnt^P1^* [51], *dfoxoΔ^94^* [6] and UAS-GFP^RNAi^ [72] were backcrossed at least 6 times into the wild-type, outbred, Dahomey population carrying the *w^1118^* mutation, which had been cured of *Wolbachia* infection over five years ago, and frequently outcrossed back into the same wild-type population. The *UAS-Aop^RNAi^* construct was obtained from the TRiP collection (HMS01256) [49], and was tracked by PCR during backcrosses as above. The Dahomey stock was collected in 1970 in Dahomey (now Benin) and has been kept in population cages maintaining its lifespan and fecundity at levels similar to freshly caught stocks. Combinations of transgenes/mutants were created using standard fly genetic techniques while avoiding population bottlenecks. To create *UAS-FLAG-Aop^ACT^*, the *Aop^ACT^* open reading frame was amplified from genomic DNA of flies carrying *UAS-Aop^ACT^* (primers are given in **Protocol S1**) and cloned into pENTR-D-TOPO vector, confirmed by sequencing and transferred to pTFW P-element-based vector, integrated into the fly genome and the transgene backcrossed as above.

The lines were maintained, and all experiments performed, at 25°C with 60% humidity and 12 h∶12 h light∶dark cycle, on sugar-yeast-agar (1SYA) food [73]. Experimental flies developed at standardised densities and once-mated females we sorted on day two of adulthood onto food containing 200 µM RU486 (Sigma) or control food as required. Lifespans were performed as described [1]. Heamolymph extraction and measurements of circulating glucose and trahalose, and other phenotyping, were performed as described [4], [74], [75]. Examination of the “smurf” phenotype was carried out using fluorescein in 1SYA +/− RU486 as described [59], except the flies were kept on the food for 18 h.

### Chromatin immunoprecipitation

Biological triplicates were done for all fly chromatin preparations. For each experiment all the batching was done so that the treatments to be compared were carried out in parallel. Chromatin was prepared from 7-day old *S_1_106>GFP-dfoxo* or *S_1_106>FLAG-Aop^ACT^* (experimental) or *S_1_106>dfoxo* or *S_1_106>Aop^ACT^* (mock control) females fed RU486-containing food from day two of adulthood, as follows: 1000 females were crushed to a fine powder under liquid nitrogen and re-suspended in 6 ml of PBS supplemented with Protease Inhibitors Cocktail (10 µl per ml, Sigma). Cross-linking was performed with 0.5% formaldehyde for 10 min and quenched with addition of 1.5 ml of 2.5 M glycine. The cross-linked chromatin was recovered by centrifugation and washed twice with FA/SDS buffer (50 mM Hepes-KOH pH 7.5, 150 mM NaCl, 1 mM EDTA, 0.1% Na deoxycholate, 0.1% SDS, 1% Triton –X-100 and 1 mM PMSF) re-suspended in the same and incubated for 1 h at 4°C. Chromatin was again recovered by centrifugation and sheared to an average size of 400 bp by sonication, giving on average 6 ml of chromatin in FA/SDS.

For anti-GFP IPs, anti-GFP antibody (Abcam, 1 µl per IP) was bound to Protein-G Dynabeads (Invitrogen) and incubated for 2 h at room temperature with 450 µl of chromatin. Beads were washed once with FA/SDS, 3 times with FA/SDS containing 500 mM NaCl, once with 10 mM Tris-HCl pH 8, 250 mM LiCl, 1 mM EDTA, 1% NP40 and 0.5% Na deoxycholate, and once with TE. For array hybridisation, the entire IP after volume reduction, or 50 ng of total chromatin DNA, were amplified two times (Whole Genome Amplification kit, Sigma) as per manufacturer's instructions. The material from the IP was hybridised against the input material. The labelling and hybridisations were carried out by Nimblegen Systems, using custom *Drosophila* whole-genome tiling arrays with probes spaced approximately every 300 bp, as described [76]. ChIP-chip data were normalized using the LIMMA package [77] in Bioconductor [78], applying loess normalization within each array and quantile normalization between arrays. Replicate information was pooled by taking the median probe value for each set of arrays and was smoothed along each chromosome using a running median within a window of three probes. Experimental signal was adjusted by mock control (pre-immune serum) data by direct subtraction of median probe intensity values. Peaks were called using the Ringo package [79] in R, using a y0 threshold of 0.97 and a distance cut-off of 600 bp.

Anti-FLAG IPs were performed essentially as above, except anti-FLAG antibody (Sigma, 3 µl per IP) was used on 1350 µl of chromatin. Subsequent processing was the same except that 100 µM dUTP was spiked in during amplification, and chromatin and IP were separately hybridized to Affymetrix Drosophila Tiling 2.0R arrays. The IP to chromatin ratios were computationally determined after normalisation using the Starr package [80], and peaks identified as above, except that smoothing was over 5 probes.

Genes were associated to peaks if any gene feature occurred within 1 kb upstream or downstream of the outermost peak probes. The overlap between regions bound by GFP-dFOXO and FLAG-AOP^ACT^ was compared to the random distribution by bootstrap analysis (10000 iterations looking for marginal basepair overlap in block bootstrap) [81] and the Z-score and p value calculated.

Chromatin was also prepared from wild-type female flies and serum-starved S2 cells and IP performed with the affinity-purified anti-dFOXO antibody [32] as described [17]. Primers used for qPCR are given in **Protocol S1**.

### Microarray and mRNA expression analysis

For microarray analysis of *dfoxo* regulated genes at least four and up to five biological repeats of 7-day old *S_1_106* or *S_1_106>dfoxo* females that had been fed RU486-containing or control food, were dissected removing either the mid-gut (50 per sample), or the abdominal/thorasic fat body (25 per sample) as associated with the cuticle, and the RNA extracted with RNeasy (Qiagen). The fat body dissections were performed by isolating and opening the fly thorax and/or abdomen, removing the internal organs with minimal disturbance of the fat body and keeping the fat body associated with the cuticle. We estimate that such dissection of the fat body results in ∼80% purity. For microarray analysis of *Aop^ACT^* regulated genes four biological repeats of 7-day old *S_1_106>Aop^ACT^* females that had been fed RU486-containing or control food, were dissected removing the abdominal fat body (25 per sample) as associated with the cuticle and the RNA extracted with RNeasy (Qiagen). All dissections and subsequent processing were done in batches so that the samples to be compared were always processed in parallel. The RNA was further processed into cRNA using standard Affymetrix protocols and hybridized to the Affymetrix Drosophila Genome 2.0 Genechip at SHWFGF/Glasgow Polyomics (University of Glasgow). The data were analysed in R. They were summarised and normalised using RMA as implemented in the LIMMA package [82]–[84]. Differential expression was assessed using linear models and the empirical Bayes moderated t-statistic implemented in LIMMA. For *dfoxo*-regulated gene analysis the effects in the linear model were: “dissection batch”, “*S_1_106*+RU486”, “*S_1_106>dfoxo*” and “*S_1_106>dfoxo*+RU486”; for *Aop^ACT^*-regulated genes: “dissection batch” and “*S_1_106>Aop^ACT^*+RU486”. Present/Absent calls were performed with Mas5 and transcripts present on at least four arrays for fat body, or at least five for gut were kept for further analysis. FDR was controlled using the described procedure [85]. For Gene Ontology enrichment, Catmap [40], or DAVID EASE [86] analyses were performed.

For qPCR analysis RNA was isolated from five to 10 midguts, five fat bodies or five guts and fat bodies with TRIZOL, converted into cDNA and qPCR performed as described [17]. The primers are given in **Protocol S1**.

### Boolean network modeling

The network was modelled using BoolNet in R [87].

### Immunofluorescence and western blots

Abdominal fat bodies as associated with the cuticle were dissected and immunofluorescence was performed using a monoclonal mouse anti-AOP antibody [48] at 1∶100 dilution using the described protocol [74]. For GFP detection, dissected body parts were fixed for 10 min before visualisation. Images were captured on Zeiss LSM 700, and quantified using ImageJ. For western blots, proteins were extracted from whole flies, or from 10 fat bodies, with TCA, or obtained by denaturing adult haemplymph in Lemmini buffer [74], separated by SDS-PAGE, transferred to nitrocellulose and probed with anti-V5 antibody (1∶500, Sigma), anti-IMP-L2 (1∶2500) [74], anti-ACTIN (1∶1000, Abcam) or anti-AOP (1∶500).

### Statistical analysis

Statistical analyses were performed in JMP (version 9) software (SAS Institute), R or Excel (Microsoft). qPCR data were log-transformed to fit a normal distribution and analysed with ANOVA, linear models or mixed effects linear models, followed by selected pair-wise comparisons using t-test. Survival analysis was performed with Log-rank, CPH or mixed-effects CPH methods in JMP, or in R using *survival* and *coxme* packages (Terry Therneau, http://CRAN.R-project.org/package=survival, http://CRAN.R-project.org/package=coxme). Analysis of smurf data was performed with a generalised linear model in R and quasibinomial distribution. Analysis of feeding was performed in JMP, using a generalised linear model, binomial distribution and over-dispersion adjustment. Significance of set overlaps was determined with Hypergeometric distribution. Details of tests performed are given in figure legends.

### Array data

The unprocessed array data are available from ArrayExpress under accession numbers: E-MTAB-1020, E-MTAB-1021, E-MTAB-927, and E-MTAB-1306.

## Supporting Information

Figure S1 **A** *dfoxo* mRNA was quantified relative to *Act* by qPCR in the guts or fat bodies of *S_1_106>dfoxo* or *TIGS>dfoxo* females, induced or not with RU486. Boxplots show log-10 derived relative expression with − RU486 values set to zero. Gut: data were analysed with a linear model and the effect of genotype was not significant (p = 0.08), while the effect of RU486 (p<10^−4^) and the interaction (p = 0.02) were significant. Each −RU486 was significantly different to its respective +RU486 condition, and the two +RU486 conditions were also significantly different (t-test, n = 3–6, p<0.05). Fat body: The −RU486 and +RU486 conditions were significantly different (t-test, n = 3–5, p = 8×10^−4^) **B** Correlation of log-transformed, scaled, raw intensity values (ratio of ChIP to input) for the three biological repeats of anti-GFP ChIP-chip performed on chromatin from *S_1_106>GFP-dfoxo* + RU486 (ChIP) or *S_1_106>dfoxo* + RU486 (mock) females for the probes within GFP-dFOXO bound peaks. **C** The sites bound by GFP-dFOXO when induced by the *S_1_106* driver in the gut and fat body were compared to the locations bound by endogenous dFOXO in whole flies [17]. **D** The distribution across genomic features of the GFP-dFOXO binding in the gut and fat body was compared to the random distribution determined from simulation of 1000 random peak sets, of identical size, length and chromosomal distribution and the Z-scores calculated. The frequency of occurrence of all the shown features was significantly different from random (p<10^−3^). Upstream and downstream refer to 1 kb from an annotated gene. “Genes” refers to regions containing annotated genes, as opposed to gene-free regions. **E** Motifs identified by MEME from the sequences bound by dFOXO in the gut and fat body (above: E value = 1.6×10^−264^, below: E value = 9.7×10^−7^) that were similar to other forkhead motifs (e.g above: Foxl1 secondary p = 2×10^−3^, below: Foxj1 primary p = 4.5×10^−7^). **F** The relative expression levels of the 5 TFs directly regulated by *dfoxo* induction in the gut. The expression levels are from the microarray experiment profiling gut mRNAs in *S_1_106>dfoxo* females with and without RU486 and are scaled to the − RU486 control for each gene. **G** The intron-containing *dfoxo* mRNA was quantified relative to *Actin* mRNA in whole *S_1_106>dfoxo* flies in presence or absence of RU486. t-test indicated significant difference between the two conditions (n = 4, p = 0.01). Note the mRNA expressed from the transgene is intron-less.
(PDF)

Figure S2 **A** *Aop* mRNA was quantified relative to *Actin* mRNA in guts and fat bodies (combined) of *S_1_106>Aop^RNAi^* flies in presence or absence of RU486. t-test indicated significant difference between the two conditions (n = 4, p = 0.04). **B** Survival of female flies expressing RNAi construct targeting *GFP* in combination with *dfoxo*. Log-Rank test detected significant differences in survival with or without RU486 for *S_1_106>GFPRNAi dfoxo* but not *S_1_106>GFPRNAi* females (*S_1_106>GFPRNAi*: p>0.05; total dead/censored: − RU486 117/1, + RU486 114/1; median/maximum lifespan: − RU486: 73/85, + RU486 76/87; *S_1_106>GFPRNAi dfoxo*: p<3×10^−3^; total dead/censored: − RU486 114/1, + RU486 136/5; median/maximum lifespan: − RU486: 78/90, + RU486 87/97). CPH analysis revealed no significant effect of RU486, significant effect of *dfoxo* (p = 7×10^−4^) and significant interaction (p = 5×10^−5^). **C** *Aop*, *dfoxo* and *Pnt* mRNA was quantified relative to *Actin* mRNA in *S_1_106>PntP1 dfoxo* and *S_1_106>PntP1 dfoxo Aop^ACT^* females in presence or absence of RU486. Boxplots show log-10 derived relative expression with − RU486 values set to zero. Data (n = 4) for each transcript were analysed with a linear model. *Aop*: There was a significant effect of genotype (p = 0.003) and significant effect of RU486 by genotype interaction (p = 0.003), where the levels in *S_1_106>PntP1 dfoxo Aop^ACT^* + RU486 were significantly different to all others (p<0.05). *dfoxo*: Only the effect of RU484 was significant (p = 0.003). *Pnt*: Only the effect of RU486 was significant (p<10^−4^). Hence, the induction of *dfoxo* and *Pnt* is not different between the genotypes.
(PDF)

Figure S3 **A** GFP expression was visualised in midguts of *S_1_106>GFP* and *TIGS>GFP* females. Red squares denote the anterior and the mid regions of the midgut were both drivers activate transgene expression. The settings used to capture images for the two drivers are the same and the GFP intensities between the two drivers can be compared. **B** Parts of the anterior midgut showing GFP expression in individual cells observed in *S_1_106>GFP* and *TIGS>GFP* females. Both drivers express in the enterocytes, which can be recognised by the intense DAPI staining and large nuclei. Note the settings used to capture the images for *S_1_106>GFP* and *TIGS>GFP* females were not the same so that the intensity of GFP cannot be compared between drivers. **C** Survival of female flies carrying *TIGS* alone in the presence or absence of RU486. Log-rank detected no significant differences (p>0.05, n≈130). **D** Survival of female flies carrying *UAS-Pnt^P1^* alone in the presence or absence of RU486. Log-rank detected no significant differences (p>0.05, n≈140).
(PDF)

Figure S4 **A** Correlation of log-transformed ratios (ChIP to input) of scaled, raw intensity values for the three biological repeats of anti-FLAG ChIP-chip performed on chromatin from *S_1_106>FLAG-Aop^ACT^* + RU486 (ChIP) or *S_1_106>Aop^ACT^* + RU486 (mock) females for the probes within FLAG-AOP^ACT^ bound peaks. **B** The distribution across genomic features of the FLAG-AOP^ACT^ binding in the gut and fat body was compared to the random distribution by bootstrap analysis [81] and the Z-scores calculated. The frequency of occurrence of all the shown features was significantly different from random (p<10^−3^). Upstream and downstream refer to 1 kb from an annotated gene. “Genes” refers to regions containing annotated genes, as opposed to gene-free regions. **C** The frequency of the lengths of regions bound by GFP-dFOXO (green) or FLAG-AOP^ACT^ (red) was plotted as smoothed density. **D** Percentage of regions bound by GFP-dFOXO or FLAG-AOP^ACT^ in adult gut/fat body that overlap the regions bound by AOP in larvae [58]. “6×” and “stage 11” refer to top 3% of the peaks from two different experiments described by Webber and colleagues [58].
(PDF)

Figure S5 **A** *lipA* mRNA was quantified relative to *Act* by qPCR in the females of the indicated genotypes, induced or not with RU486. Boxplots show log-10 derived relative expression with − RU486 values set to 0. Data were analysed with a linear model and the effects of RU486, genotype and their interaction were significant (n = 4, p<10^−3^), however, the *S_1_106>dfoxo Pnt^P1^* + RU486 condition was not significantly different from the *S_1_106>dfoxo* + RU486 condition (t-test , p = 0.2). **B** The proportion of “smurfs” (flies with impaired gut barrier function, n = 80–95) in the noted genotypes after 3 weeks of RU486 feeding, or the − RU486 condition. Data were analysed with a generalised linear model and quasibinomial distribution, and no significant effect of RU486, genotype or their interaction was detected (p>0.25). **C** Proportion of *S_1_106>dfoxo Pnt^P1^* females feeding after 5 days of feeding on RU486 or control food. Data were analysed with a generalised linear model, binomial distribution adjusted for over-dispersion, and no significant effect of RU486 was detected (p = 0.1). **D** Starvation survival of *S_1_106> Pnt^P1^ dfoxo* and *S_1_106> Pnt^P1^ dfoxo Aop^ACT^* females after 5 days of feeding on RU486 or the control food. RU486 significantly reduced the survival of *S_1_106> Pnt^P1^ dfoxo* females (Log-rank, n∼100, p = 4×10^−4^) but not of the *S_1_106> Pnt^P1^ dfoxo Aop^ACT^* females (Log-rank, n∼100, p = 0.4). CHP analysis detected a significant effect of RU486 (p = 9×10^−3^), but marginal effect of genotype (p = 0.06) and marginal RU486 by genotype interaction (p = 0.08).
(PDF)

Figure S6 **A** Survival of *S_1_106*-alone control females in the absence or presence of RU486. Log-rank test detected no significant differences (p>0.05, n≈150). **B** Survival of *UAS-Aop^ACT^*-alone control females in the absence or presence of RU486. Log-rank test detected no significant differences (p>0.05, n≈150). **C** Lifespan of *S_1_106>FLAG-Aop^ACT^* female flies in presence or absence of RU486. The two conditions were different by Log-rank test (p = 3×10^−5^; total dead/censored: − RU486 143/4, + RU486 147/0; median/maximum lifespan: − RU486: 71/83, + RU486 76/87). **D** *Aop* mRNA was quantified relative to *Act* by qPCR in the guts of *S_1_106>Aop^ACT^* or *TIGS>Aop^ACT^* females, induced or not with RU486. Boxplots show log-10 derived relative expression with −RU486 values set to zero. Data (n = 2–4) were analysed with a linear model and the effects of genotype, RU486 and their interaction were all significant(p<10^−4^). Each + RU486 was significantly different to its respective − RU486 condition, and the two + RU486 conditions were also significantly different (t-test, p<0.05).
(PDF)

Figure S7 *S_1_106>Aop^ACT^* female flies were placed on appropriate food on day two and either frozen on day 7 for metabolic assays or kept for stress and fecundity assays. For stress assays, 7-day old females were placed on food containing H_2_O_2_ (no yeast) or DDT, or were starved on food containing agar alone, and the number of dead flies scored over time. There were no significant differences in survival by Long-rank test (n≈100, p>0.05) in any of the conditions. Levels of trahalose were determined as number of moles of trehalose per fly weight, of lipids as weight of TAG per fly weight, of glycogen as weight of glycogen per fly weight and all are shown as percentage of no-RU486 control. There were no significant differences by t-test (n = 10, p>0.05). Eggs laid per female per day (averaged per vial of 10 females) were determined once per week for the first 4 weeks and summed to give an estimate of lifetime fecundity. There were no significant differences (t-test, n = 10, p>0.05). Feeding was assessed in 8-day old females and no significant differences were detected.
(PDF)

Figure S8 **A** *Aop* mRNA (left) or AOP protein (right) levels were determined against *Act*/ACT control by qPCR or western blots in the fat bodies of *S_1_106>Aop^ACT^* females fed RU486 or control food. qPCR data were log-10 derived and significant difference found between −RU486 and + RU486 conditions (t-test, p = 8×10^−4^). **B** Quantification of the results presented in Figure 8A. AOP was visualised by immunofuorescence in fat bodies of *S_1_106>Aop^ACT^* flies induced or not with RU486. Intensity of nuclear AOP staining was quantified from confocal images using Image J (intensity is presented on an arbitrary scale). Average intensity from at least 3 cells from each animal was calculated for each biological repeat, and location of the nucleus was determined from DAPI staining. For + RU486 samples, the nuclei were binned into “high” or “low” AOP stained ones by visual inspection and quantified separately. Means ± SEM are shown. n = 3 animals − RU486, n = 4 for + RU486; t-test detected a significant difference (p<10^−4^) between the “high AOP” nuclei on + RU486 and nuclei on − RU486. The proportion of highly stained nuclei was 26%+/−6% in 4 animals fed RU486 examined. **C** *S_1_106* drives expression in all the cells of the fat body. *S_1_106>GFP* flies were fed or not RU486 from day two until day 7 when GFP expression in the fat body was determined by confocal microscopy. GFP was detected uniformly in all the cells of the abdominal fat body.
(PDF)

Figure S9 **A** Biological process GO categories differentially regulated (p<10^−10^) in the fat body upon induction of *dfoxo* or *Aop^ACT^* as determined by Catmap analysis. Any redundant categories (overlap by more than 75%) were removed, retaining the most specific category. The full list is given in **Dataset S1**. The intensity of red shows the log_10_-transformed p-value associated with differential regulation for each category. **B** Median lifespan extension caused by induction or *dfoxo*, *Aop^ACT^* or both with RU486 and the *S_1_106* driver in two independent experimental trials. In each case RU486 had a significant, positive effect on survival (Log-rank, p<10^−3^). The survival data (n = 1664 deaths/23 censors) were analysed with a mixed effects CPH model, with experimental trial as random effect, and the effect of genotype (p<0.05) and RU486 (p = 10^−12^) were significant but their interaction was not (p>0.1), revealing that the two factors do not have additive effects.
(PDF)

Dataset S1 Lists of genes and genomic locations and full Ease and Catmap results mentioned in the manuscript.
(ZIP)

Protocol S1 Sequences of primers used in this study.
(PDF)

## References

[1] Giannakou ME, Goss M, Junger MA, Hafen E, Leevers SJ, et al (2004) Long-lived Drosophila with overexpressed dFOXO in adult fat body. Science 305: 361. 15192154 10.1126/science.1098219

[2] Hwangbo DS, Gershman B, Tu MP, Palmer M, Tatar M (2004) Drosophila dFOXO controls lifespan and regulates insulin signalling in brain and fat body. Nature 429: 562–566. 15175753 10.1038/nature02549

[3] Demontis F, Perrimon N (2010) FOXO/4E-BP signaling in Drosophila muscles regulates organism-wide proteostasis during aging. Cell 143: 813–825. 21111239 10.1016/j.cell.2010.10.007 PMC3066043

[4] Alic N, Tullet J, Niccoli T, Broughton S, Hoddinott MP, et al (2014) Cell-nonautonomous effects of dFOXO/DAF-16 in ageing. Cell Rep 6: 608–616. 24508462 10.1016/j.celrep.2014.01.015 PMC3969275

[5] Kenyon C, Chang J, Gensch E, Rudner A, Tabtiang R (1993) A C. elegans mutant that lives twice as long as wild type. Nature 366: 461–464. 8247153 10.1038/366461a0

[6] Slack C, Giannakou ME, Foley A, Goss M, Partridge L (2011) dFOXO-independent effects of reduced insulin-like signaling in Drosophila. Aging Cell 10: 735–748. 21443682 10.1111/j.1474-9726.2011.00707.x PMC3193374

[7] Postnikoff SD, Malo ME, Wong B, Harkness TA (2012) The yeast forkhead transcription factors fkh1 and fkh2 regulate lifespan and stress response together with the anaphase-promoting complex. PLoS Genet 8: e1002583. 22438832 10.1371/journal.pgen.1002583 PMC3305399

[8] Kuningas M, Magi R, Westendorp RG, Slagboom PE, Remm M, et al (2007) Haplotypes in the human Foxo1a and Foxo3a genes; impact on disease and mortality at old age. Eur J Hum Genet 15: 294–301. 17245409 10.1038/sj.ejhg.5201766

[9] Willcox BJ, Donlon TA, He Q, Chen R, Grove JS, et al (2008) FOXO3A genotype is strongly associated with human longevity. Proc Natl Acad Sci U S A 105: 13987–13992. 18765803 10.1073/pnas.0801030105 PMC2544566

[10] Flachsbart F, Caliebe A, Kleindorp R, Blanche H, von Eller-Eberstein H, et al (2009) Association of FOXO3A variation with human longevity confirmed in German centenarians. Proc Natl Acad Sci U S A 106: 2700–2705. 19196970 10.1073/pnas.0809594106 PMC2650329

[11] Anselmi CV, Malovini A, Roncarati R, Novelli V, Villa F, et al (2009) Association of the FOXO3A locus with extreme longevity in a southern Italian centenarian study. Rejuvenation Res 12: 95–104. 19415983 10.1089/rej.2008.0827

[12] Pawlikowska L, Hu D, Huntsman S, Sung A, Chu C, et al (2009) Association of common genetic variation in the insulin/IGF1 signaling pathway with human longevity. Aging Cell 8: 460–472. 19489743 10.1111/j.1474-9726.2009.00493.x PMC3652804

[13] Eijkelenboom A, Burgering BM (2013) FOXOs: signalling integrators for homeostasis maintenance. Nat Rev Mol Cell Biol 14: 83–97. 23325358 10.1038/nrm3507

[14] Murphy CT, McCarroll SA, Bargmann CI, Fraser A, Kamath RS, et al (2003) Genes that act downstream of DAF-16 to influence the lifespan of Caenorhabditis elegans. Nature 424: 277–283. 12845331 10.1038/nature01789

[15] McElwee JJ, Schuster E, Blanc E, Piper MD, Thomas JH, et al (2007) Evolutionary conservation of regulated longevity assurance mechanisms. Genome Biol 8: R132. 17612391 10.1186/gb-2007-8-7-r132 PMC2323215

[16] Schuster E, McElwee JJ, Tullet JM, Doonan R, Matthijssens F, et al (2010) DamID in C. elegans reveals longevity-associated targets of DAF-16/FoxO. Mol Syst Biol 6: 399. 20706209 10.1038/msb.2010.54 PMC2950082

[17] Alic N, Andrews TD, Giannakou ME, Papatheodorou I, Slack C, et al (2011) Genome-wide dFOXO targets and topology of the transcriptomic response to stress and insulin signalling. Mol Syst Biol 7: 502. 21694719 10.1038/msb.2011.36 PMC3159968

[18] Bai H, Ping K, Hernandez AM, Tatar M (2013) Activin Signaling Targeted by Insulin/dFOXO Regulates Aging and Muscle Proteostasis in Drosophila. PLoS Genet 9 (11) e1003941. 24244197 10.1371/journal.pgen.1003941 PMC3820802

[19] Webb AE, Pollina EA, Vierbuchen T, Urban N, Ucar D, et al (2013) FOXO3 shares common targets with ASCL1 genome-wide and inhibits ASCL1-dependent neurogenesis. Cell Rep 4: 477–491. 23891001 10.1016/j.celrep.2013.06.035 PMC3838667

[20] Eijkelenboom A, Mokry M, de Wit E, Smits LM, Polderman PE, et al (2013) Genome-wide analysis of FOXO3 mediated transcription regulation through RNA polymerase II profiling. Mol Syst Biol 9: 638. 23340844 10.1038/msb.2012.74 PMC3564262

[21] Puig O, Tjian R (2005) Transcriptional feedback control of insulin receptor by dFOXO/FOXO1. Genes Dev 19: 2435–2446. 16230533 10.1101/gad.1340505 PMC1257398

[22] van der Vos KE, Coffer PJ (2008) FOXO-binding partners: it takes two to tango. Oncogene 27: 2289–2299. 18391971 10.1038/onc.2008.22

[23] Bouchard C, Marquardt J, Bras A, Medema RH, Eilers M (2004) Myc-induced proliferation and transformation require Akt-mediated phosphorylation of FoxO proteins. EMBO J 23: 2830–2840. 15241468 10.1038/sj.emboj.7600279 PMC514943

[24] Nemoto S, Fergusson MM, Finkel T (2004) Nutrient availability regulates SIRT1 through a forkhead-dependent pathway. Science 306: 2105–2108. 15604409 10.1126/science.1101731

[25] Ogg S, Paradis S, Gottlieb S, Patterson GI, Lee L, et al (1997) The Fork head transcription factor DAF-16 transduces insulin-like metabolic and longevity signals in C. elegans. Nature 389: 994–999. 9353126 10.1038/40194

[26] Seoane J, Le HV, Shen L, Anderson SA, Massague J (2004) Integration of Smad and forkhead pathways in the control of neuroepithelial and glioblastoma cell proliferation. Cell 117: 211–223. 15084259 10.1016/s0092-8674(04)00298-3

[27] Essers MA, de Vries-Smits LM, Barker N, Polderman PE, Burgering BM, et al (2005) Functional interaction between beta-catenin and FOXO in oxidative stress signaling. Science 308: 1181–1184. 15905404 10.1126/science.1109083

[28] Teleman AA, Hietakangas V, Sayadian AC, Cohen SM (2008) Nutritional control of protein biosynthetic capacity by insulin via Myc in Drosophila. Cell Metab 7: 21–32. 18177722 10.1016/j.cmet.2007.11.010

[29] Paik JH, Kollipara R, Chu G, Ji H, Xiao Y, et al (2007) FoxOs are lineage-restricted redundant tumor suppressors and regulate endothelial cell homeostasis. Cell 128: 309–323. 17254969 10.1016/j.cell.2006.12.029 PMC1855089

[30] Tothova Z, Kollipara R, Huntly BJ, Lee BH, Castrillon DH, et al (2007) FoxOs are critical mediators of hematopoietic stem cell resistance to physiologic oxidative stress. Cell 128: 325–339. 17254970 10.1016/j.cell.2007.01.003

[31] Poirier L, Shane A, Zheng J, Seroude L (2008) Characterization of the Drosophila gene-switch system in aging studies: a cautionary tale. Aging Cell 7: 758–770. 18691185 10.1111/j.1474-9726.2008.00421.x

[32] Giannakou ME, Goss M, Jacobson J, Vinti G, Leevers SJ, et al (2007) Dynamics of the action of dFOXO on adult mortality in Drosophila. Aging Cell 6: 429–438. 17465980 10.1111/j.1474-9726.2007.00290.x

[33] Giannakou ME, Goss M, Partridge L (2008) Role of dFOXO in lifespan extension by dietary restriction in Drosophila melanogaster: not required, but its activity modulates the response. Aging Cell 7: 187–198. 18241326 10.1111/j.1474-9726.2007.00362.x

[34] Min KJ, Yamamoto R, Buch S, Pankratz M, Tatar M (2008) Drosophila lifespan control by dietary restriction independent of insulin-like signaling. Aging Cell 7: 199–206. 18221413 10.1111/j.1474-9726.2008.00373.x PMC2340190

[35] Libina N, Berman JR, Kenyon C (2003) Tissue-specific activities of C. elegans DAF-16 in the regulation of lifespan. Cell 115: 489–502. 14622602 10.1016/s0092-8674(03)00889-4

[36] Bluher M, Kahn BB, Kahn CR (2003) Extended longevity in mice lacking the insulin receptor in adipose tissue. Science 299: 572–574. 12543978 10.1126/science.1078223

[37] Puig O, Marr MT, Ruhf ML, Tjian R (2003) Control of cell number by Drosophila FOXO: downstream and feedback regulation of the insulin receptor pathway. Genes Dev 17: 2006–2020. 12893776 10.1101/gad.1098703 PMC196255

[38] Junger MA, Rintelen F, Stocker H, Wasserman JD, Vegh M, et al (2003) The Drosophila forkhead transcription factor FOXO mediates the reduction in cell number associated with reduced insulin signaling. J Biol 2: 20. 12908874 10.1186/1475-4924-2-20 PMC333403

[39] Karpac J, Biteau B, Jasper H (2013) Misregulation of an adaptive metabolic response contributes to the age-related disruption of lipid homeostasis in Drosophila. Cell Rep 4: 1250–1261. 24035390 10.1016/j.celrep.2013.08.004 PMC3832190

[40] Breslin T, Eden P, Krogh M (2004) Comparing functional annotation analyses with Catmap. BMC Bioinformatics 5: 193. 15588298 10.1186/1471-2105-5-193 PMC543458

[41] Bahadorani S, Cho J, Lo T, Contreras H, Lawal HO, et al (2010) Neuronal expression of a single-subunit yeast NADH-ubiquinone oxidoreductase (Ndi1) extends Drosophila lifespan. Aging Cell 9: 191–202. 20089120 10.1111/j.1474-9726.2010.00546.x PMC2860002

[42] Sanz A, Soikkeli M, Portero-Otin M, Wilson A, Kemppainen E, et al (2010) Expression of the yeast NADH dehydrogenase Ndi1 in Drosophila confers increased lifespan independently of dietary restriction. Proc Natl Acad Sci U S A 107: 9105–9110. 20435911 10.1073/pnas.0911539107 PMC2889079

[43] Casas-Tinto S, Marr MT 2nd, Andreu P, Puig O (2007) Characterization of the Drosophila insulin receptor promoter. Biochim Biophys Acta 1769: 236–243. 17462750 10.1016/j.bbaexp.2007.03.003

[44] Roukens MG, Alloul-Ramdhani M, Moghadasi S, Op den Brouw M, Baker DA (2008) Downregulation of vertebrate Tel (ETV6) and Drosophila Yan is facilitated by an evolutionarily conserved mechanism of F-box-mediated ubiquitination. Mol Cell Biol 28: 4394–4406. 18426905 10.1128/MCB.01914-07 PMC2447152

[45] Roukens MG, Alloul-Ramdhani M, Baan B, Kobayashi K, Peterson-Maduro J, et al (2010) Control of endothelial sprouting by a Tel-CtBP complex. Nat Cell Biol 12: 933–942. 20835243 10.1038/ncb2096

[46] Hock H, Meade E, Medeiros S, Schindler JW, Valk PJ, et al (2004) Tel/Etv6 is an essential and selective regulator of adult hematopoietic stem cell survival. Genes Dev 18: 2336–2341. 15371326 10.1101/gad.1239604 PMC522982

[47] Lai ZC, Rubin GM (1992) Negative control of photoreceptor development in Drosophila by the product of the yan gene, an ETS domain protein. Cell 70: 609–620. 1505027 10.1016/0092-8674(92)90430-k

[48] Rebay I, Rubin GM (1995) Yan functions as a general inhibitor of differentiation and is negatively regulated by activation of the Ras1/MAPK pathway. Cell 81: 857–866. 7781063 10.1016/0092-8674(95)90006-3

[49] Ni JQ, Zhou R, Czech B, Liu LP, Holderbaum L, et al (2011) A genome-scale shRNA resource for transgenic RNAi in Drosophila. Nat Methods 8: 405–407. 21460824 10.1038/nmeth.1592 PMC3489273

[50] Cox DR (1972) Regression models and life-tables. J R Stat Soc Ser B Stat Methodol 34: 187–220.

[51] O'Neill EM, Rebay I, Tjian R, Rubin GM (1994) The activities of two Ets-related transcription factors required for Drosophila eye development are modulated by the Ras/MAPK pathway. Cell 78: 137–147. 8033205 10.1016/0092-8674(94)90580-0

[52] Brunner D, Ducker K, Oellers N, Hafen E, Scholz H, et al (1994) The ETS domain protein pointed-P2 is a target of MAP kinase in the sevenless signal transduction pathway. Nature 370: 386–389. 8047146 10.1038/370386a0

[53] Halfon MS, Carmena A, Gisselbrecht S, Sackerson CM, Jimenez F, et al (2000) Ras pathway specificity is determined by the integration of multiple signal-activated and tissue-restricted transcription factors. Cell 103: 63–74. 11051548 10.1016/s0092-8674(00)00105-7

[54] Rera M, Bahadorani S, Cho J, Koehler CL, Ulgherait M, et al (2011) Modulation of longevity and tissue homeostasis by the Drosophila PGC-1 homolog. Cell Metab 14: 623–634. 22055505 10.1016/j.cmet.2011.09.013 PMC3238792

[55] Buchon N, Osman D, David FP, Fang HY, Boquete JP, et al (2013) Morphological and molecular characterization of adult midgut compartmentalization in Drosophila. Cell Rep 3: 1725–1738. 23643535 10.1016/j.celrep.2013.04.001

[56] Marianes A, Spradling AC (2013) Physiological and stem cell compartmentalization within the Drosophila midgut. Elife 2: e00886. 23991285 10.7554/eLife.00886 PMC3755342

[57] Negre N, Brown CD, Ma L, Bristow CA, Miller SW, et al (2011) A cis-regulatory map of the Drosophila genome. Nature 471: 527–531. 21430782 10.1038/nature09990 PMC3179250

[58] Webber JL, Zhang J, Cote L, Vivekanand P, Ni X, et al (2013) The relationship between long-range chromatin occupancy and polymerization of the Drosophila ETS family transcriptional repressor Yan. Genetics 193: 633–649. 23172856 10.1534/genetics.112.146647 PMC3567750

[59] Rera M, Clark RI, Walker DW (2012) Intestinal barrier dysfunction links metabolic and inflammatory markers of aging to death in Drosophila. Proc Natl Acad Sci U S A 109: 21528–21533. 23236133 10.1073/pnas.1215849110 PMC3535647

[60] Zhang W, Thompson BJ, Hietakangas V, Cohen SM (2011) MAPK/ERK signaling regulates insulin sensitivity to control glucose metabolism in Drosophila. PLoS Genet 7: e1002429. 22242005 10.1371/journal.pgen.1002429 PMC3248469

[61] Brunet A, Bonni A, Zigmond MJ, Lin MZ, Juo P, et al (1999) Akt promotes cell survival by phosphorylating and inhibiting a Forkhead transcription factor. Cell 96: 857–868. 10102273 10.1016/s0092-8674(00)80595-4

[62] Rothemund S, Liou YC, Davies PL, Krause E, Sonnichsen FD (1999) A new class of hexahelical insect proteins revealed as putative carriers of small hydrophobic ligands. Structure 7: 1325–1332. 10574794 10.1016/s0969-2126(00)80022-2

[63] Graham LA, Davies PL (2002) The odorant-binding proteins of Drosophila melanogaster: annotation and characterization of a divergent gene family. Gene 292: 43–55. 12119098 10.1016/s0378-1119(02)00672-8

[64] Gronke S, Clarke D-F, Broughton S, Andrews TD, Partridge L (2010) Molecular evolution and functional characterisation of Drosophila insulin-like peptides. PLoS Genet 6: e1000857. 20195512 10.1371/journal.pgen.1000857 PMC2829060

[65] Broughton SJ, Piper MD, Ikeya T, Bass TM, Jacobson J, et al (2005) Longer lifespan, altered metabolism, and stress resistance in Drosophila from ablation of cells making insulin-like ligands. Proc Natl Acad Sci U S A 102: 3105–3110. 15708981 10.1073/pnas.0405775102 PMC549445

[66] Lemmon MA, Schlessinger J (2010) Cell signaling by receptor tyrosine kinases. Cell 141: 1117–1134. 20602996 10.1016/j.cell.2010.06.011 PMC2914105

[67] Rogge R, Green PJ, Urano J, Horn-Saban S, Mlodzik M, et al (1995) The role of yan in mediating the choice between cell division and differentiation. Development 121: 3947–3958. 8575295 10.1242/dev.121.12.3947

[68] Fontana L, Partridge L, Longo VD (2010) Extending healthy life span–from yeast to humans. Science 328: 321–326. 20395504 10.1126/science.1172539 PMC3607354

[69] Gudbjartsson DF, Walters GB, Thorleifsson G, Stefansson H, Halldorsson BV, et al (2008) Many sequence variants affecting diversity of adult human height. Nat Genet 40: 609–615. 18391951 10.1038/ng.122

[70] Lango Allen H, Estrada K, Lettre G, Berndt SI, Weedon MN, et al (2010) Hundreds of variants clustered in genomic loci and biological pathways affect human height. Nature 467: 832–838. 20881960 10.1038/nature09410 PMC2955183

[71] Fujii S, Amrein H (2002) Genes expressed in the Drosophila head reveal a role for fat cells in sex-specific physiology. EMBO J 21: 5353–5363. 12374736 10.1093/emboj/cdf556 PMC129088

[72] Roignant JY, Carre C, Mugat B, Szymczak D, Lepesant JA, et al (2003) Absence of transitive and systemic pathways allows cell-specific and isoform-specific RNAi in Drosophila. RNA 9: 299–308. 12592004 10.1261/rna.2154103 PMC1370397

[73] Bass TM, Grandison RC, Wong R, Martinez P, Partridge L, et al (2007) Optimization of dietary restriction protocols in Drosophila. J Gerontol A Biol Sci Med Sci 62: 1071–1081. 17921418 10.1093/gerona/62.10.1071 PMC4335187

[74] Alic N, Hoddinott MP, Vinti G, Partridge L (2011) Lifespan extension by increased expression of the Drosophila homologue of the IGFBP7 tumour suppressor. Aging Cell 10: 137–147. 21108726 10.1111/j.1474-9726.2010.00653.x PMC3042147

[75] Broughton S, Alic N, Slack C, Bass T, Ikeya T, et al (2008) Reduction of DILP2 in Drosophila triages a metabolic phenotype from lifespan revealing redundancy and compensation among DILPs. PLoS One 3: e3721. 19005568 10.1371/journal.pone.0003721 PMC2579582

[76] Choksi SP, Southall TD, Bossing T, Edoff K, de Wit E, et al (2006) Prospero acts as a binary switch between self-renewal and differentiation in Drosophila neural stem cells. Dev Cell 11: 775–789. 17141154 10.1016/j.devcel.2006.09.015

[77] Smyth GK, Speed T (2003) Normalization of cDNA microarray data. Methods 31: 265–273. 14597310 10.1016/s1046-2023(03)00155-5

[78] Gentleman RC, Carey VJ, Bates DM, Bolstad B, Dettling M, et al (2004) Bioconductor: open software development for computational biology and bioinformatics. Genome Biol 5: R80. 15461798 10.1186/gb-2004-5-10-r80 PMC545600

[79] Toedling J, Skylar O, Krueger T, Fischer JJ, Sperling S, et al (2007) Ringo–an R/Bioconductor package for analyzing ChIP-chip readouts. BMC Bioinformatics 8: 221. 17594472 10.1186/1471-2105-8-221 PMC1906858

[80] Zacher B, Kuan PF, Tresch A (2010) Starr: Simple Tiling ARRay analysis of Affymetrix ChIP-chip data. BMC Bioinformatics 11: 194. 20398407 10.1186/1471-2105-11-194 PMC2868012

[81] Bickel P, Boley N, Brown J, Huang H, Zhang N (2010) Subsampling Methods for Genomic Inference. Ann Appl Stat 4: 1660–1697.

[82] Bolstad BM, Irizarry RA, Astrand M, Speed TP (2003) A comparison of normalization methods for high density oligonucleotide array data based on variance and bias. Bioinformatics 19: 185–193. 12538238 10.1093/bioinformatics/19.2.185

[83] Irizarry RA, Bolstad BM, Collin F, Cope LM, Hobbs B, et al (2003) Summaries of Affymetrix GeneChip probe level data. Nucleic Acids Res 31: e15. 12582260 10.1093/nar/gng015 PMC150247

[84] Irizarry RA, Hobbs B, Collin F, Beazer-Barclay YD, Antonellis KJ, et al (2003) Exploration, normalization, and summaries of high density oligonucleotide array probe level data. Biostatistics 4: 249–264. 12925520 10.1093/biostatistics/4.2.249

[85] Benjamini Y, Hochberg Y (1995) Controlling the False Discovery Rate: A practical and Powerful Approach to Multiple Testing. J R Stat Soc Ser B Stat Methodol 57: 289–300.

[86] Huang da W, Sherman BT, Lempicki RA (2009) Systematic and integrative analysis of large gene lists using DAVID bioinformatics resources. Nat Protoc 4: 44–57. 19131956 10.1038/nprot.2008.211

[87] Mussel C, Hopfensitz M, Kestler HA (2010) BoolNet–an R package for generation, reconstruction and analysis of Boolean networks. Bioinformatics 26: 1378–1380. 20378558 10.1093/bioinformatics/btq124

