# Supplementary figures and images for: Interplay of dFOXO and Two ETS-Family Transcription Factors Determines Lifespan in Drosophila melanogaster
Source: PLoS Genet. 2014 Sep 18;10(9):e1004619. doi: 10.1371/journal.pgen.1004619 (PMC4169242; doi:10.1371/journal.pgen.1004619)

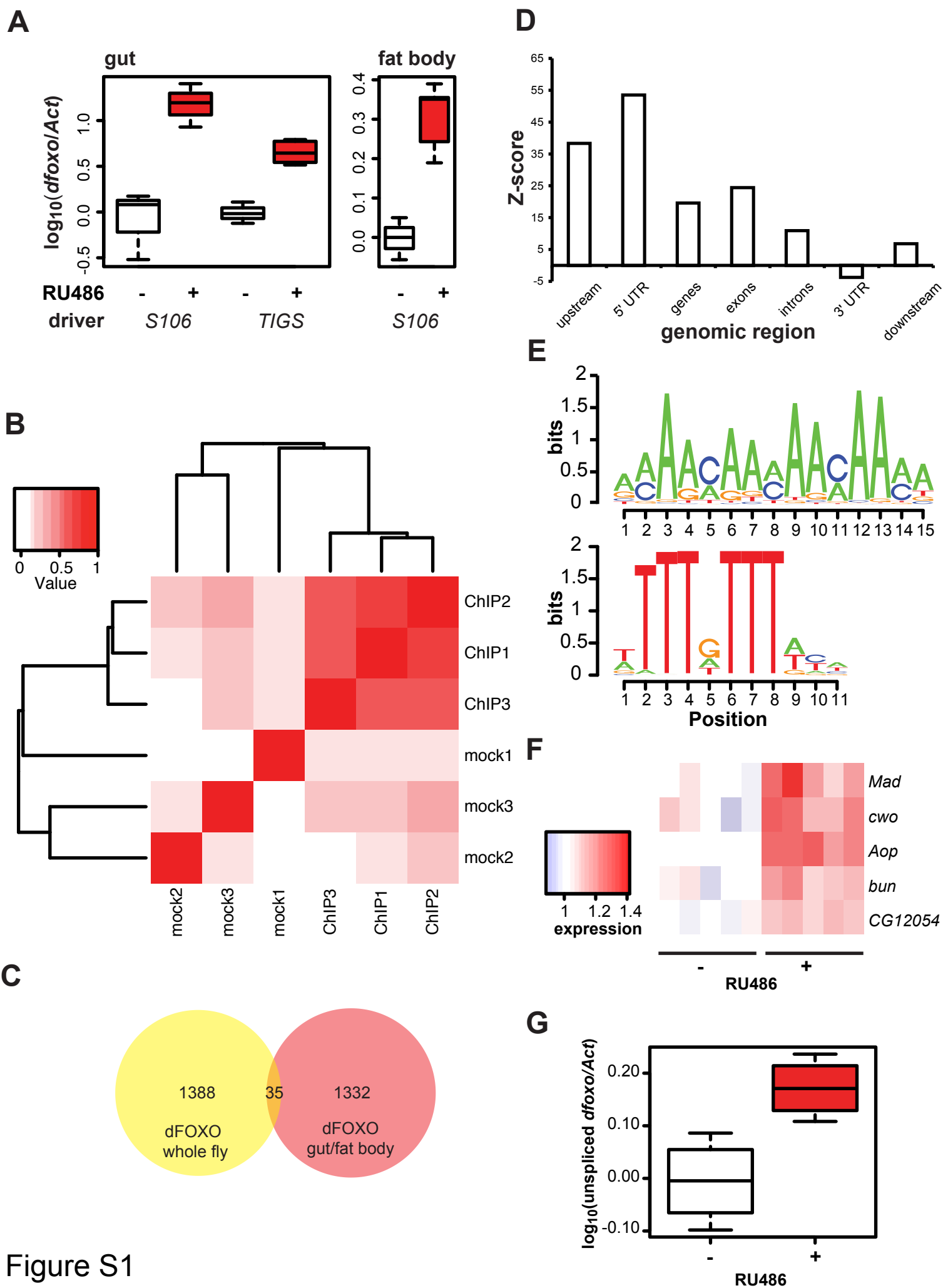

Figure S1

Supplement: Figure S1 — A dfoxo mRNA was quantified relative to Act by qPCR in the guts or fat bodies of S1106>dfoxo or TIGS>dfoxo females, induced or not with RU486. Boxplots show log-10 derived relative expression with − RU486 values set to zero. Gut: data were analysed with a linear model and the effect of genotype was not significant (p = 0.08), while the effect of RU486 (p<10−4) and the interaction (p = 0.02) were significant. Each −RU486 was significantly different to its respective +RU486 condition, and the two +RU486 conditions were also significantly different (t-test, n = 3–6, p<0.05). Fat body: The −RU486 and +RU486 conditions were significantly different (t-test, n = 3–5, p = 8×10^−4^) B Correlation of log-transformed, scaled, raw intensity values (ratio of ChIP to input) for the three biological repeats of anti-GFP ChIP-chip performed on chromatin from S1106>GFP-dfoxo + RU486 (ChIP) or S1106>dfoxo + RU486 (mock) females for the probes within GFP-dFOXO bound peaks. C The sites bound by GFP-dFOXO when induced by the S1106 driver in the gut and fat body were compared to the locations bound by endogenous dFOXO in whole flies [17]. D The distribution across genomic features of the GFP-dFOXO binding in the gut and fat body was compared to the random distribution determined from simulation of 1000 random peak sets, of identical size, length and chromosomal distribution and the Z-scores calculated. The frequency of occurrence of all the shown features was significantly different from random (p<10−3). Upstream and downstream refer to 1 kb from an annotated gene. “Genes” refers to regions containing annotated genes, as opposed to gene-free regions. E Motifs identified by MEME from the sequences bound by dFOXO in the gut and fat body (above: E value = 1.6×10−264, below: E value = 9.7×10^−7^) that were similar to other forkhead motifs (e.g above: Foxl1 secondary p = 2×10^−3^, below: Foxj1 primary p = 4.5×10^−7^). F The relative expression levels of the 5 TFs directly regulated by dfoxo induction [file pgen.1004619.s001.pdf]

**A**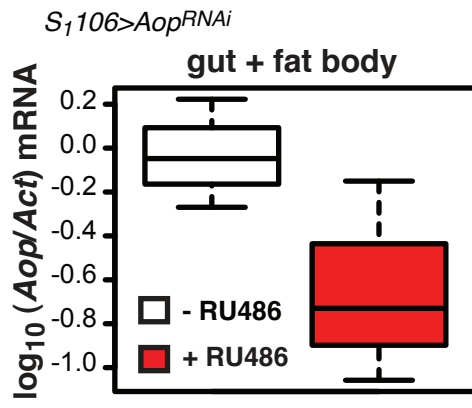**B**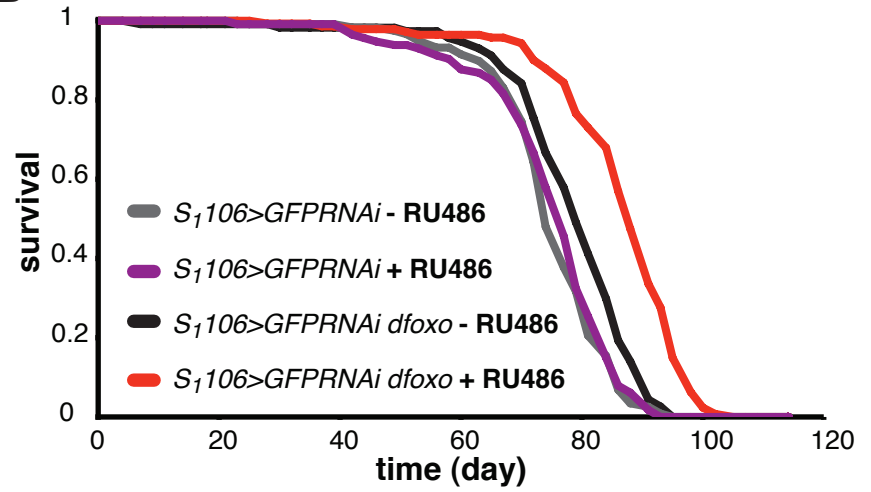**C**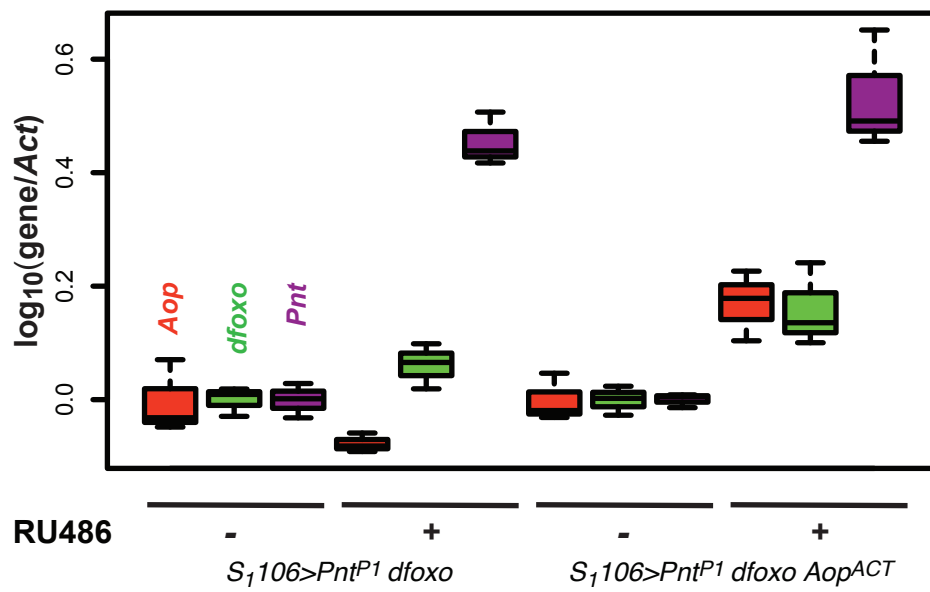

Figure S2

Supplement: Figure S2 — A Aop mRNA was quantified relative to Actin mRNA in guts and fat bodies (combined) of S1106>AopRNAi flies in presence or absence of RU486. t-test indicated significant difference between the two conditions (n = 4, p = 0.04). B Survival of female flies expressing RNAi construct targeting GFP in combination with dfoxo. Log-Rank test detected significant differences in survival with or without RU486 for S1106>GFPRNAi dfoxo but not S1106>GFPRNAi females (S1106>GFPRNAi: p>0.05; total dead/censored: − RU486 117/1, + RU486 114/1; median/maximum lifespan: − RU486: 73/85, + RU486 76/87; S1106>GFPRNAi dfoxo: p<3×10^−3^; total dead/censored: − RU486 114/1, + RU486 136/5; median/maximum lifespan: − RU486: 78/90, + RU486 87/97). CPH analysis revealed no significant effect of RU486, significant effect of dfoxo (p = 7×10^−4^) and significant interaction (p = 5×10^−5^). C Aop, dfoxo and Pnt mRNA was quantified relative to Actin mRNA in S1106>PntP1 dfoxo and S1106>PntP1 dfoxo AopACT females in presence or absence of RU486. Boxplots show log-10 derived relative expression with − RU486 values set to zero. Data (n = 4) for each transcript were analysed with a linear model. Aop: There was a significant effect of genotype (p = 0.003) and significant effect of RU486 by genotype interaction (p = 0.003), where the levels in S1106>PntP1 dfoxo AopACT + RU486 were significantly different to all others (p<0.05). dfoxo: Only the effect of RU484 was significant (p = 0.003). Pnt: Only the effect of RU486 was significant (p<10−4). Hence, the induction of dfoxo and Pnt is not different between the genotypes. (PDF) [file pgen.1004619.s002.pdf]

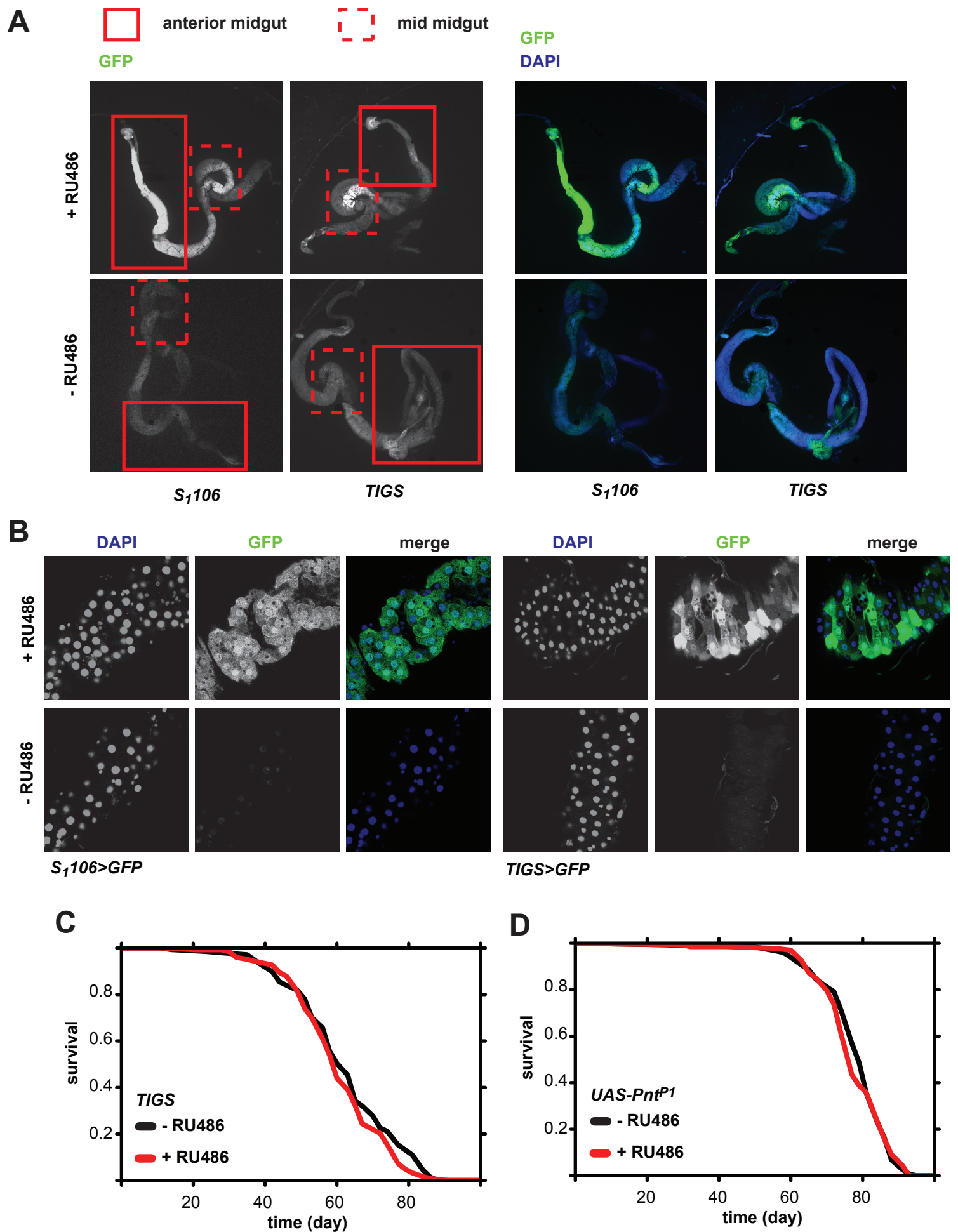

Figure S3

Supplement: Figure S3 — A GFP expression was visualised in midguts of S1106>GFP and TIGS>GFP females. Red squares denote the anterior and the mid regions of the midgut were both drivers activate transgene expression. The settings used to capture images for the two drivers are the same and the GFP intensities between the two drivers can be compared. B Parts of the anterior midgut showing GFP expression in individual cells observed in S1106>GFP and TIGS>GFP females. Both drivers express in the enterocytes, which can be recognised by the intense DAPI staining and large nuclei. Note the settings used to capture the images for S1106>GFP and TIGS>GFP females were not the same so that the intensity of GFP cannot be compared between drivers. C Survival of female flies carrying TIGS alone in the presence or absence of RU486. Log-rank detected no significant differences (p>0.05, n≈130). D Survival of female flies carrying UAS-PntP1 alone in the presence or absence of RU486. Log-rank detected no significant differences (p>0.05, n≈140). (PDF) [file pgen.1004619.s003.pdf]

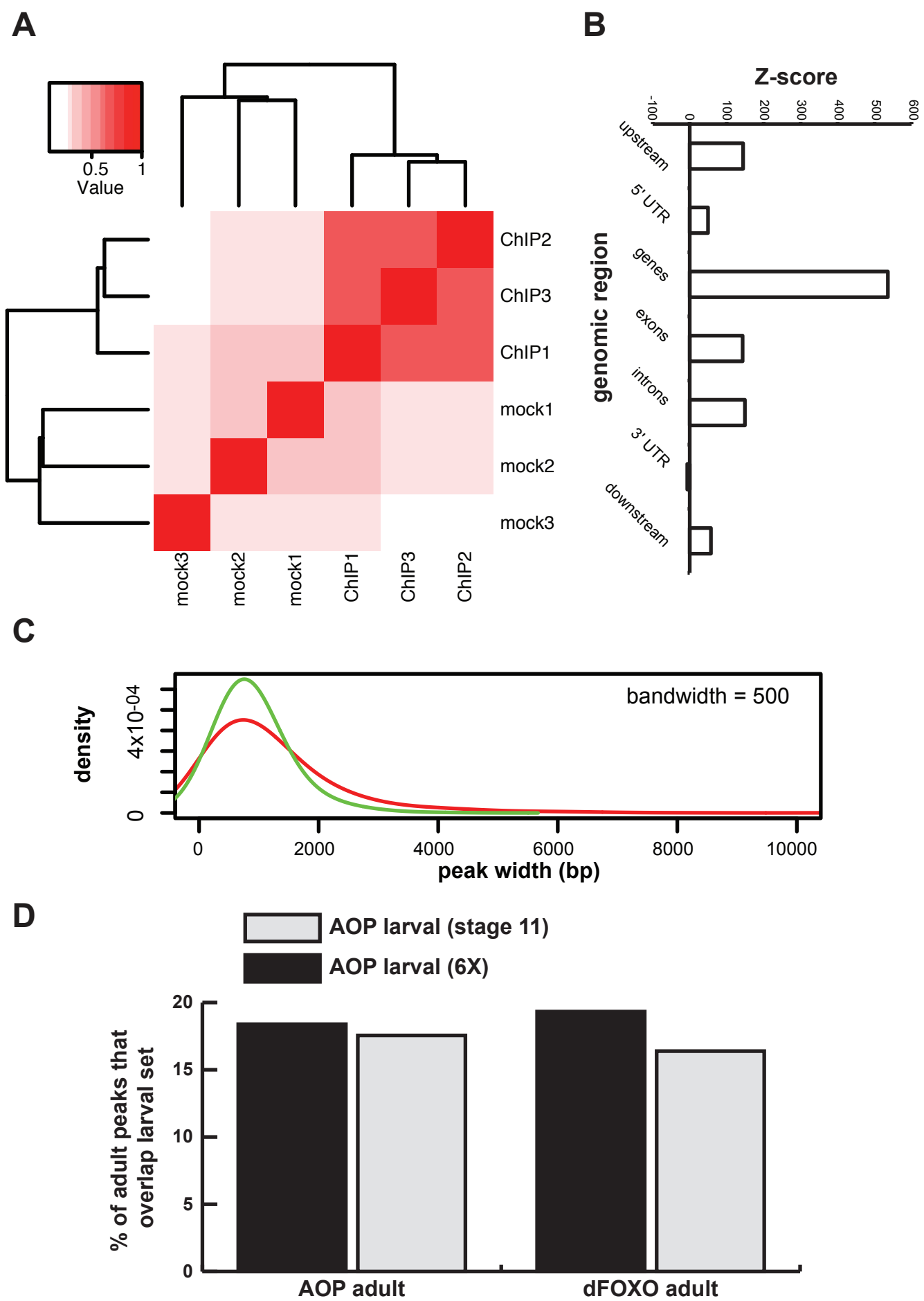

Figure S4

Supplement: Figure S4 — A Correlation of log-transformed ratios (ChIP to input) of scaled, raw intensity values for the three biological repeats of anti-FLAG ChIP-chip performed on chromatin from S1106>FLAG-AopACT + RU486 (ChIP) or S1106>AopACT + RU486 (mock) females for the probes within FLAG-AOPACT bound peaks. B The distribution across genomic features of the FLAG-AOPACT binding in the gut and fat body was compared to the random distribution by bootstrap analysis [81] and the Z-scores calculated. The frequency of occurrence of all the shown features was significantly different from random (p<10−3). Upstream and downstream refer to 1 kb from an annotated gene. “Genes” refers to regions containing annotated genes, as opposed to gene-free regions. C The frequency of the lengths of regions bound by GFP-dFOXO (green) or FLAG-AOPACT (red) was plotted as smoothed density. D Percentage of regions bound by GFP-dFOXO or FLAG-AOPACT in adult gut/fat body that overlap the regions bound by AOP in larvae [58]. “6×” and “stage 11” refer to top 3% of the peaks from two different experiments described by Webber and colleagues [58]. (PDF) [file pgen.1004619.s004.pdf]

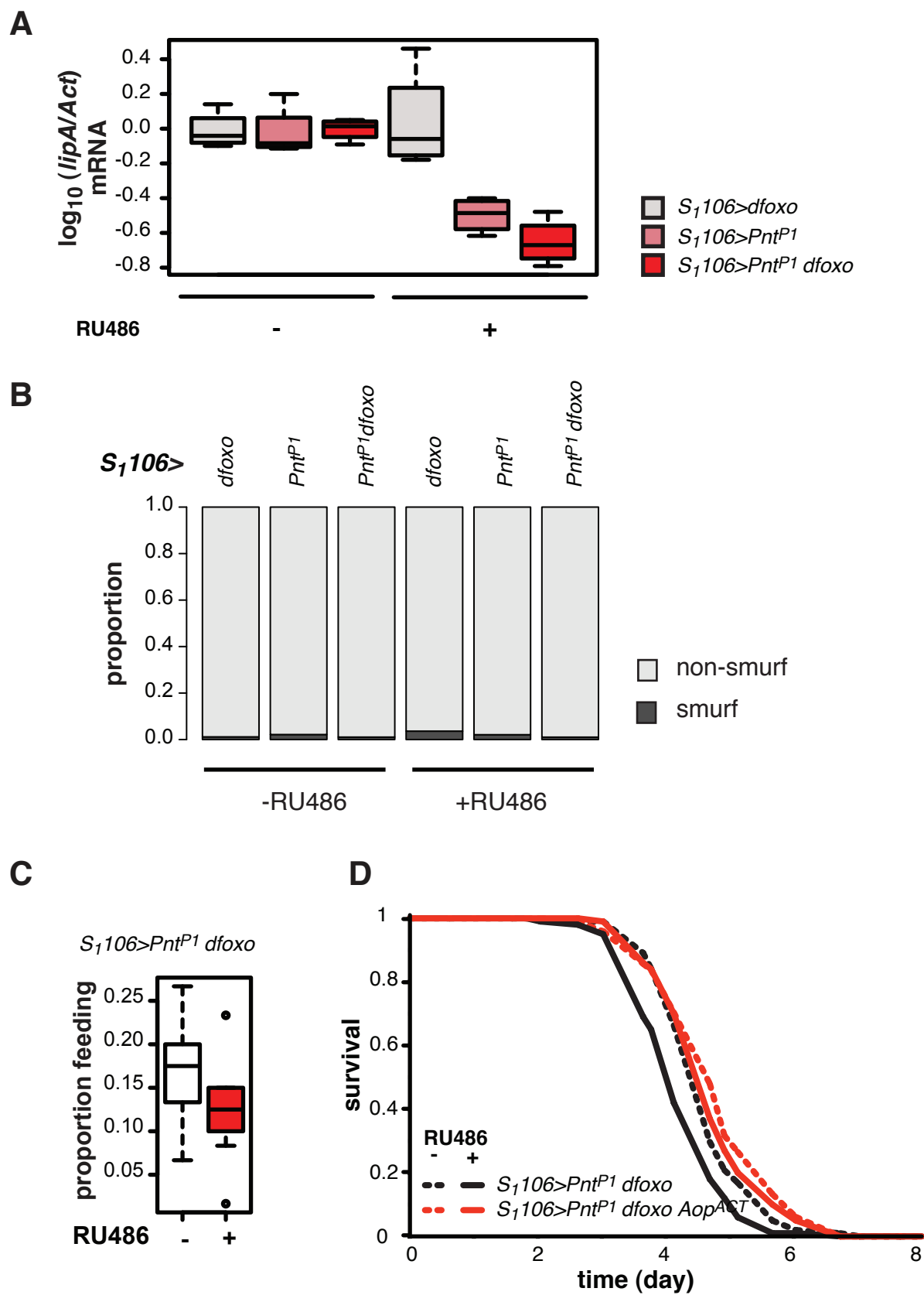

Figure S5

Supplement: Figure S5 — A lipA mRNA was quantified relative to Act by qPCR in the females of the indicated genotypes, induced or not with RU486. Boxplots show log-10 derived relative expression with − RU486 values set to 0. Data were analysed with a linear model and the effects of RU486, genotype and their interaction were significant (n = 4, p<10−3), however, the S1106>dfoxo PntP1 + RU486 condition was not significantly different from the S1106>dfoxo + RU486 condition (t-test , p = 0.2). B The proportion of “smurfs” (flies with impaired gut barrier function, n = 80–95) in the noted genotypes after 3 weeks of RU486 feeding, or the − RU486 condition. Data were analysed with a generalised linear model and quasibinomial distribution, and no significant effect of RU486, genotype or their interaction was detected (p>0.25). C Proportion of S1106>dfoxo PntP1 females feeding after 5 days of feeding on RU486 or control food. Data were analysed with a generalised linear model, binomial distribution adjusted for over-dispersion, and no significant effect of RU486 was detected (p = 0.1). D Starvation survival of S1106> PntP1 dfoxo and S1106> PntP1 dfoxo AopACT females after 5 days of feeding on RU486 or the control food. RU486 significantly reduced the survival of S1106> PntP1 dfoxo females (Log-rank, n∼100, p = 4×10^−4^) but not of the S1106> PntP1 dfoxo AopACT females (Log-rank, n∼100, p = 0.4). CHP analysis detected a significant effect of RU486 (p = 9×10^−3^), but marginal effect of genotype (p = 0.06) and marginal RU486 by genotype interaction (p = 0.08). (PDF) [file pgen.1004619.s005.pdf]

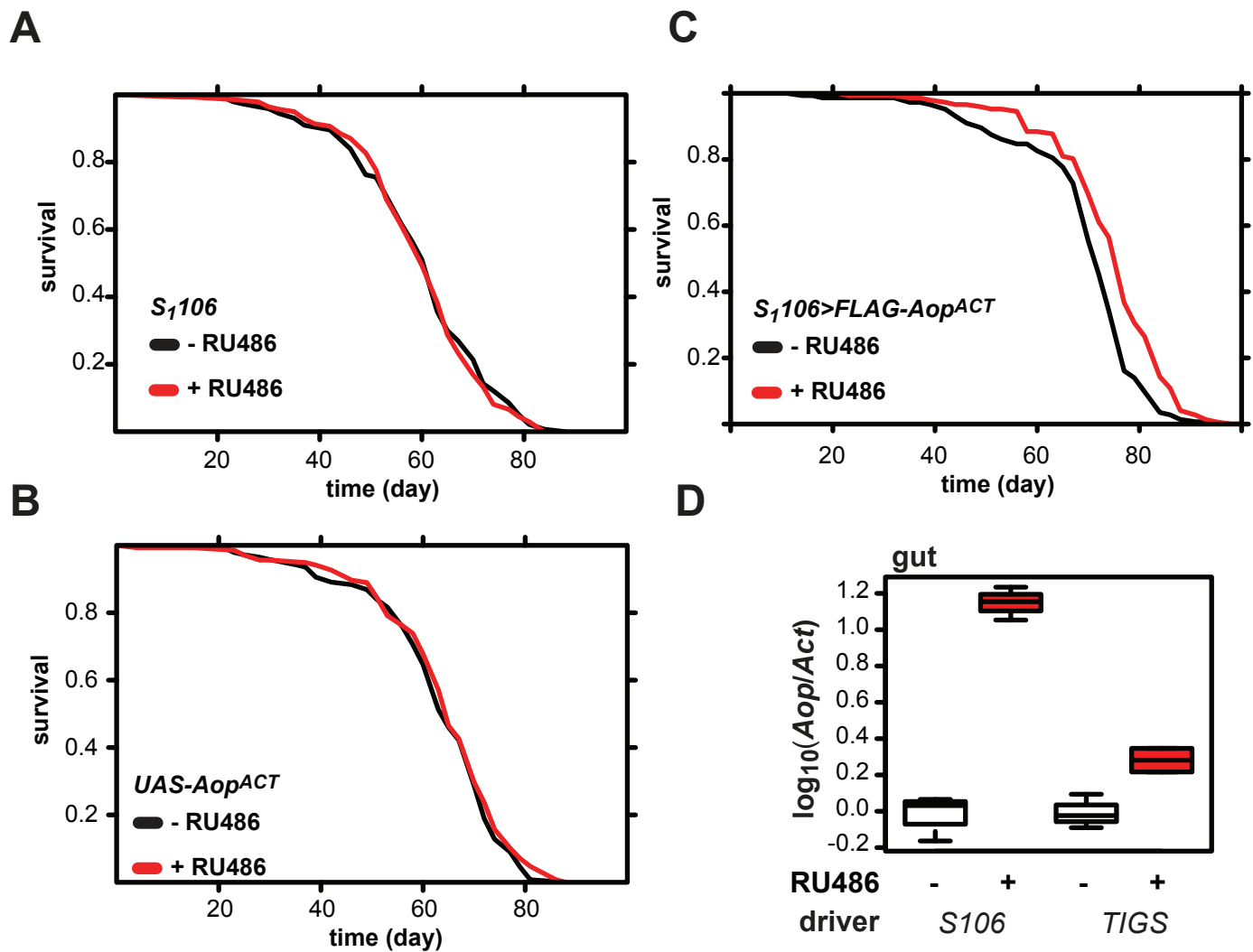

Figure S6

Supplement: Figure S6 — A Survival of S1106-alone control females in the absence or presence of RU486. Log-rank test detected no significant differences (p>0.05, n≈150). B Survival of UAS-AopACT-alone control females in the absence or presence of RU486. Log-rank test detected no significant differences (p>0.05, n≈150). C Lifespan of S1106>FLAG-AopACT female flies in presence or absence of RU486. The two conditions were different by Log-rank test (p = 3×10^−5^; total dead/censored: − RU486 143/4, + RU486 147/0; median/maximum lifespan: − RU486: 71/83, + RU486 76/87). D Aop mRNA was quantified relative to Act by qPCR in the guts of S1106>AopACT or TIGS>AopACT females, induced or not with RU486. Boxplots show log-10 derived relative expression with −RU486 values set to zero. Data (n = 2–4) were analysed with a linear model and the effects of genotype, RU486 and their interaction were all significant(p<10−4). Each + RU486 was significantly different to its respective − RU486 condition, and the two + RU486 conditions were also significantly different (t-test, p<0.05). (PDF) [file pgen.1004619.s006.pdf]

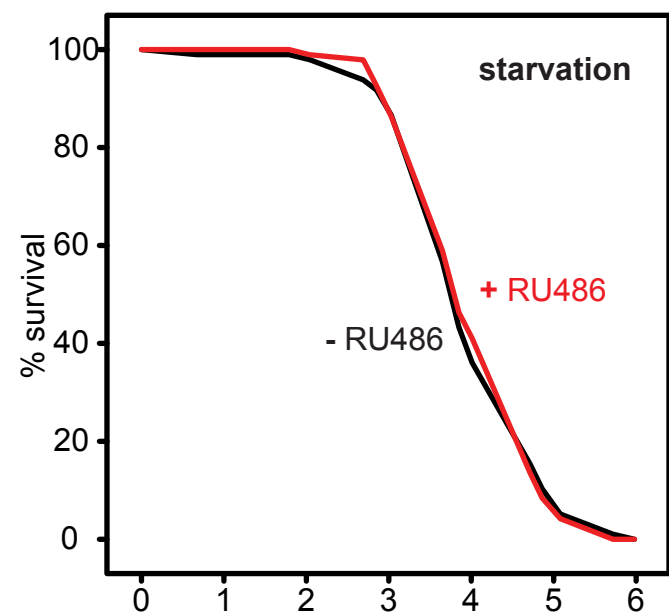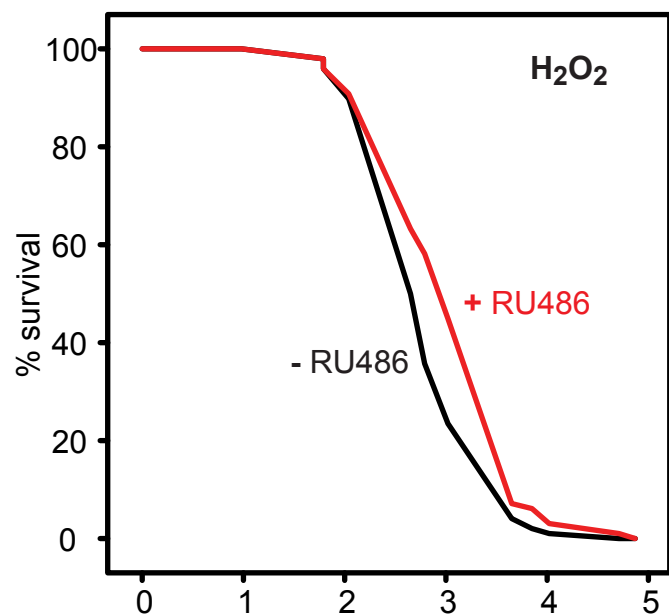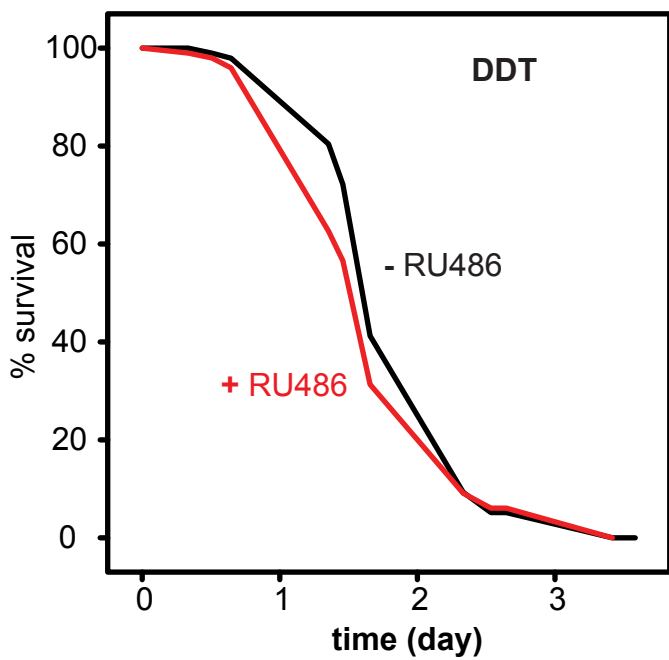

**feeding**

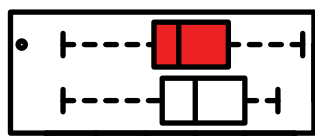

RU486

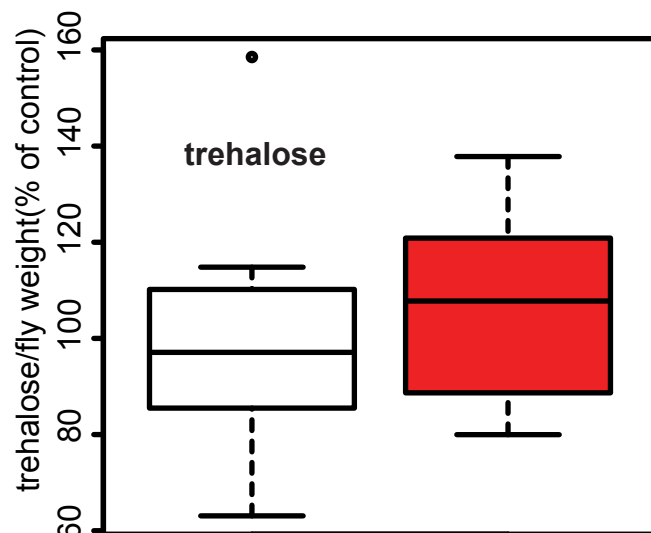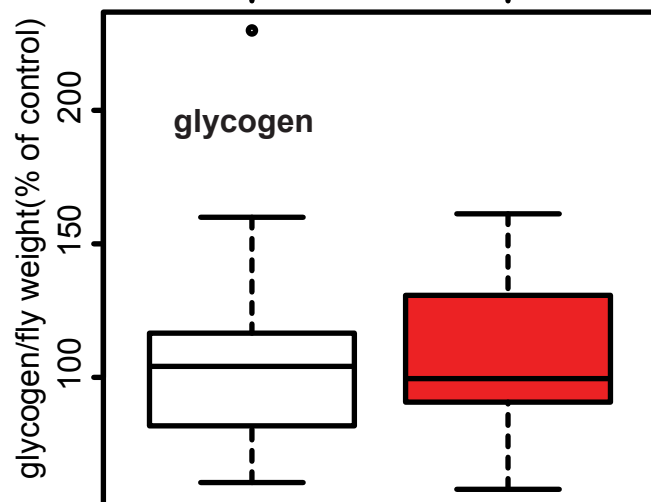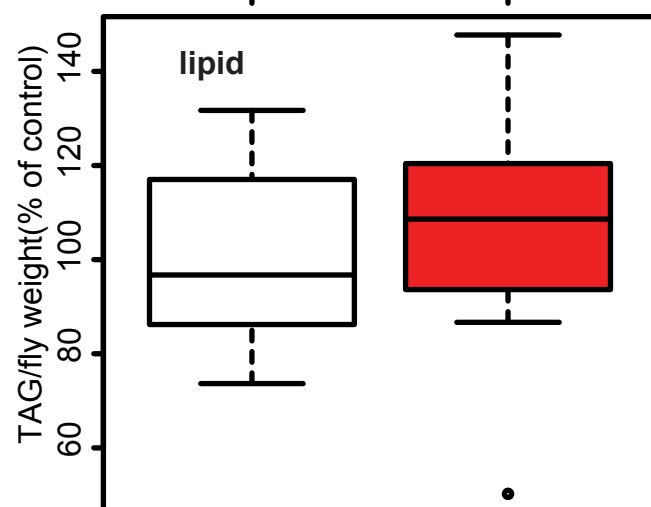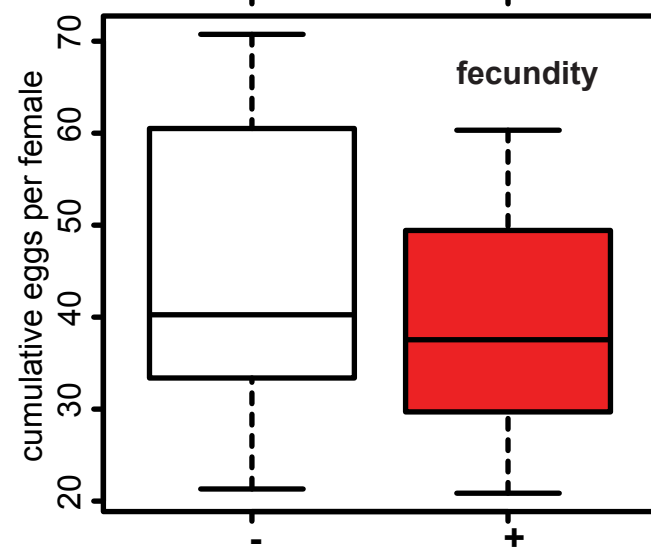

RU486

Figure S7

Supplement: Figure S7 — S1106>AopACT female flies were placed on appropriate food on day two and either frozen on day 7 for metabolic assays or kept for stress and fecundity assays. For stress assays, 7-day old females were placed on food containing H2O2 (no yeast) or DDT, or were starved on food containing agar alone, and the number of dead flies scored over time. There were no significant differences in survival by Long-rank test (n≈100, p>0.05) in any of the conditions. Levels of trahalose were determined as number of moles of trehalose per fly weight, of lipids as weight of TAG per fly weight, of glycogen as weight of glycogen per fly weight and all are shown as percentage of no-RU486 control. There were no significant differences by t-test (n = 10, p>0.05). Eggs laid per female per day (averaged per vial of 10 females) were determined once per week for the first 4 weeks and summed to give an estimate of lifetime fecundity. There were no significant differences (t-test, n = 10, p>0.05). Feeding was assessed in 8-day old females and no significant differences were detected. (PDF) [file pgen.1004619.s007.pdf]

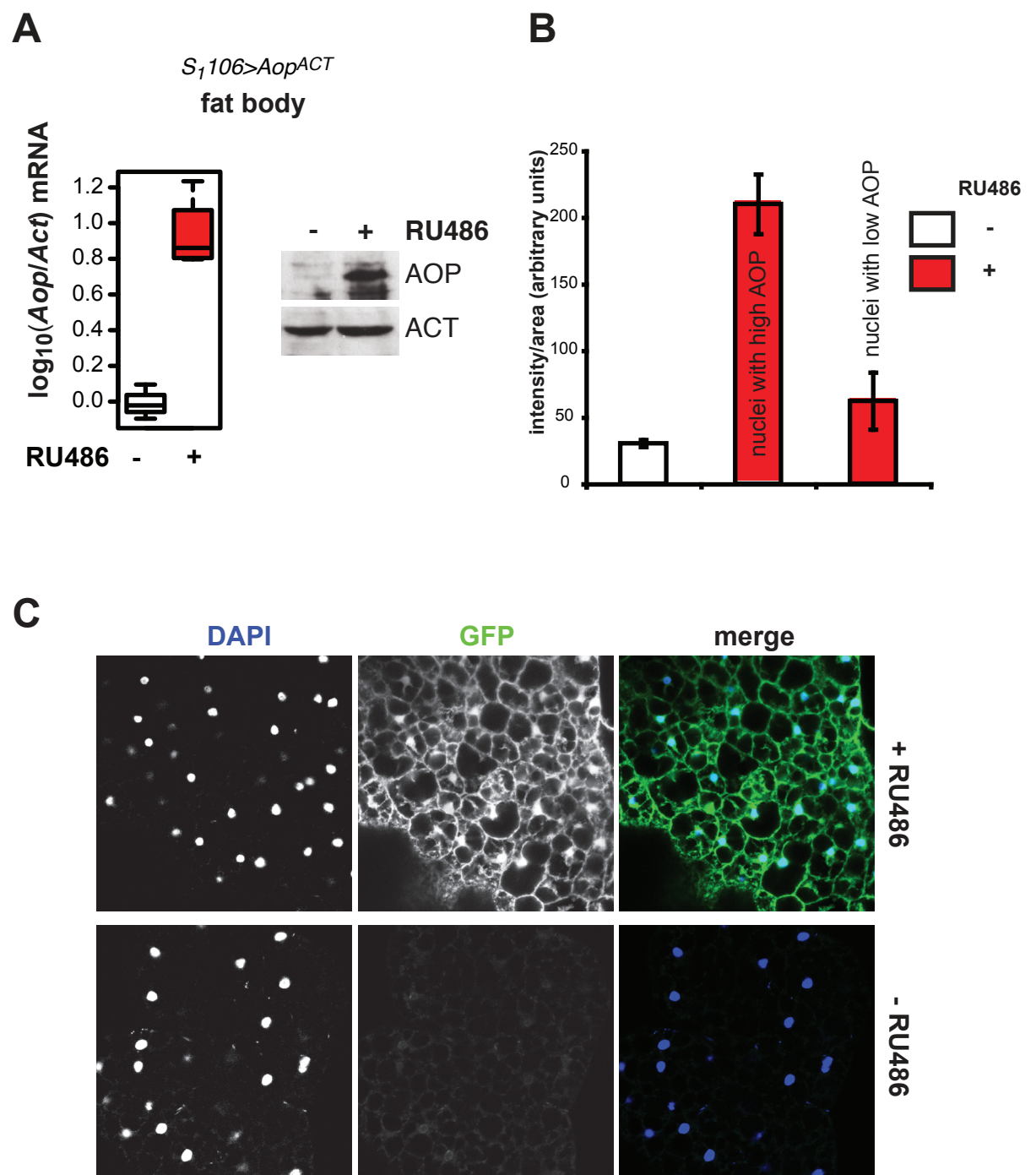

Figure S8

Supplement: Figure S8 — A Aop mRNA (left) or AOP protein (right) levels were determined against Act/ACT control by qPCR or western blots in the fat bodies of S1106>AopACT females fed RU486 or control food. qPCR data were log-10 derived and significant difference found between −RU486 and + RU486 conditions (t-test, p = 8×10^−4^). B Quantification of the results presented in Figure 8A. AOP was visualised by immunofuorescence in fat bodies of S1106>AopACT flies induced or not with RU486. Intensity of nuclear AOP staining was quantified from confocal images using Image J (intensity is presented on an arbitrary scale). Average intensity from at least 3 cells from each animal was calculated for each biological repeat, and location of the nucleus was determined from DAPI staining. For + RU486 samples, the nuclei were binned into “high” or “low” AOP stained ones by visual inspection and quantified separately. Means ± SEM are shown. n = 3 animals − RU486, n = 4 for + RU486; t-test detected a significant difference (p<10−4) between the “high AOP” nuclei on + RU486 and nuclei on − RU486. The proportion of highly stained nuclei was 26%+/−6% in 4 animals fed RU486 examined. C S1106 drives expression in all the cells of the fat body. S1106>GFP flies were fed or not RU486 from day two until day 7 when GFP expression in the fat body was determined by confocal microscopy. GFP was detected uniformly in all the cells of the abdominal fat body. (PDF) [file pgen.1004619.s008.pdf]

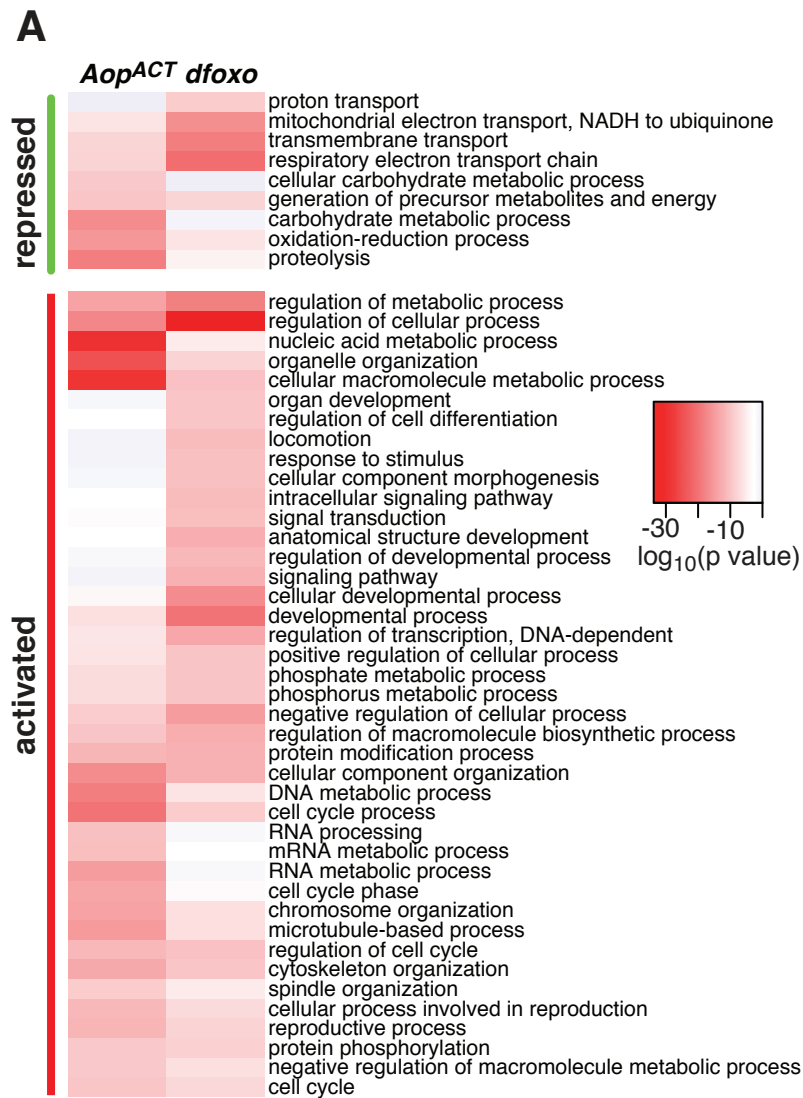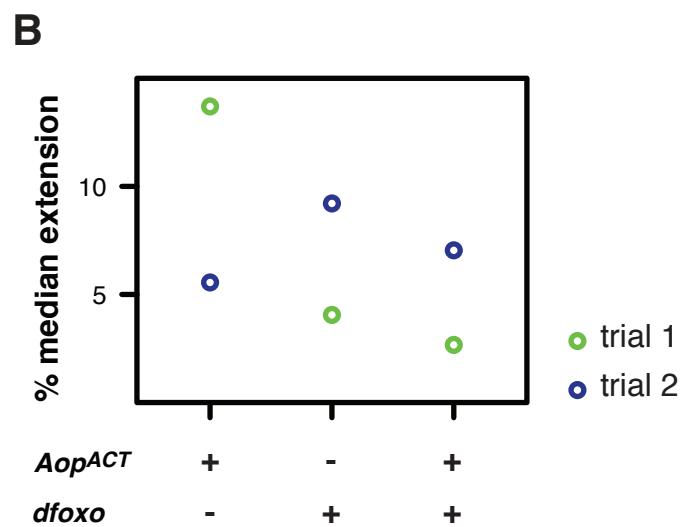

Figure S9

Supplement: Figure S9 — A Biological process GO categories differentially regulated (p<10−10) in the fat body upon induction of dfoxo or AopACT as determined by Catmap analysis. Any redundant categories (overlap by more than 75%) were removed, retaining the most specific category. The full list is given in Dataset S1. The intensity of red shows the log10-transformed p-value associated with differential regulation for each category. B Median lifespan extension caused by induction or dfoxo, AopACT or both with RU486 and the S1106 driver in two independent experimental trials. In each case RU486 had a significant, positive effect on survival (Log-rank, p<10−3). The survival data (n = 1664 deaths/23 censors) were analysed with a mixed effects CPH model, with experimental trial as random effect, and the effect of genotype (p<0.05) and RU486 (p = 10−12) were significant but their interaction was not (p>0.1), revealing that the two factors do not have additive effects. (PDF) [file pgen.1004619.s009.pdf]
