# Supplementary material for: Interplay of dFOXO and Two ETS-Family Transcription Factors Determines Lifespan in Drosophila melanogaster
Source: PLoS Genet. 2014 Sep 18;10(9):e1004619. doi: 10.1371/journal.pgen.1004619 (PMC4169242; doi:10.1371/journal.pgen.1004619)
Supplement: Protocol S1 — Sequences of primers used in this study. (PDF) [file pgen.1004619.s011.pdf]

Protocol S1

Primer Sequences

<u>Primers for ChIP analysis:</u>
InR P3
forward: GCGATGATCGCTGGAAGGTT
reverse: GCGTTTGTCAATTCCGAGGAT

Aop 3'
forward: CTTTCTTTGCGGCTGTCAAAC
reverse: TGTTGGTTTATGCAGATGTG

Aop 5'
forward: CTCGGTGTGTGTTTACATTG
reverse: TGCTCTCGCTGATGTTGCTG

U6
forward: GACTCAGTTCGTATATATAG
reverse: GGGCGCCAGTGCTCACTACT

<u>Primers used for cDNA analysis:</u>
Aop
forward: ACGAGGACTGTTATCCACTC
reverse: CTGCCTGCTGGTCATCGTG

Obp99b
forward: GTTGAGGTGCACGAATCGGAC
reverse: AGGCCTTCGTGCAGGAGTCGC

lipA
forward: CGTTAATGCCAATGCGAGC
reverse: GGATGCCCTGGTTGCTAG

CG6295
forward: GTCCATCCACTCCATGGTAC
reverse: TTGTGTCCTGGCGGCTAATG

dfoxo intronic
forward: CCACCATGCTCGCTGGTCTC
reverse: CTCCATTTGACTGACTGACTTG

Actin5C:
forward: CACACCAAATCTTACAAAATGTGTGA
reverse: AATCCGGCCTTGCACATG

Primers used for *Aop*^{ACT} cloning:
forward: CACCATGTCCAAAATGAAAATGCTCCCAG
reverse: CTACTGCTGCATGTGTCGGAAG
